# Developmental regulation of DNA cytosine methylation at the immunoglobulin heavy chain constant locus

**DOI:** 10.1371/journal.pgen.1007930

**Published:** 2019-02-19

**Authors:** Chloé Oudinet, Fatima-Zohra Braikia, Audrey Dauba, Joana M. Santos, Ahmed Amine Khamlichi

**Affiliations:** Institut de Pharmacologie et de Biologie Structurale, IPBS, Université de Toulouse, CNRS, UPS, Toulouse, France; MRC Laboratory of Molecular Biology, UNITED KINGDOM

## Abstract

DNA cytosine methylation is involved in the regulation of gene expression during development and its deregulation is often associated with disease. Mammalian genomes are predominantly methylated at CpG dinucleotides. Unmethylated CpGs are often associated with active regulatory sequences while methylated CpGs are often linked to transcriptional silencing. Previous studies on CpG methylation led to the notion that transcription initiation is more sensitive to CpG methylation than transcriptional elongation. The immunoglobulin heavy chain (*IgH*) constant locus comprises multiple inducible constant genes and is expressed exclusively in B lymphocytes. The developmental B cell stage at which methylation patterns of the *IgH* constant genes are established, and the role of CpG methylation in their expression, are unknown. Here, we find that methylation patterns at most *cis*-acting elements of the *IgH* constant genes are established and maintained independently of B cell activation or promoter activity. Moreover, one of the promoters, but not the enhancers, is hypomethylated in sperm and early embryonic cells, and is targeted by different demethylation pathways, including AID, UNG, and ATM pathways. Combined, the data suggest that, rather than being prominently involved in the regulation of the *IgH* constant locus expression, DNA methylation may primarily contribute to its epigenetic pre-marking.

## Author summary

DNA methylation mainly occurs at CpG dinucleotides and strongly influences gene expression during development. Deregulation of DNA methylation is often associated with disease. In mammalian genomes, unmethylated CpG dinucleotides are generally associated with active regulatory sequences, while methylated CpGs are often associated with silent promoters. The immunoglobulin heavy chain constant locus comprises multiple inducible constant genes and is expressed exclusively in B lymphocytes. We show that methylation patterns of most of the locus *cis*-elements, including promoters, enhancers and insulators, are established and faithfully maintained independently of B cell activation or transcription initiation. Acquisition of DNA methylation by the constant genes exons occurs independently of transcriptional elongation. One late B cell specific promoter is hypomethylated early in ontogeny. Constant genes promoters recruit different demethylation pathways that become dispensable for the maintenance of the mark in the B cell lineage. The data suggest that, rather than playing a prominent role in transcriptional regulation, DNA methylation may contribute to the epigenetic pre-marking of the *IgH* constant locus.

## Introduction

DNA methylation is a common epigenetic regulation mechanism in vertebrates and is involved in gene expression regulation during development and differentiation as well as in defense of the genome against transposable elements. DNA methylation provides a robust epigenetic mechanism for cell fate decisions, cell identity and tissue homeostasis. The importance of this epigenetic regulation is highlighted by the finding that its absence is lethal and aberrant DNA cytosine methylation is often associated with disease such as cancer [1].

Mammalian genomes are predominantly methylated at cytosines in the context of CpG dinucleotide. Mammalian genomes are mostly CpG-poor and these CpG motifs are globally methylated. However, a minority of CpGs occur in CpG-dense regions called CpG islands (CGIs) and are generally refractory to DNA methylation. While unmethylated CpG sites and CGIs are generally associated with active promoters, methylated CpGs (mCpGs) and mCGIs are closely associated with transcriptionally silent promoters. This pattern is less obvious when it comes to transcription elongation as mCpGs and mCGIs in gene body did not block elongation, leading to the notion that it is transcription initiation that is more sensitive to cytosine methylation [2–4].

B lymphocytes are derived from pluripotent hematopoietic stem cells and develop in fetal liver during embryonic development, then shift to the bone marrow around birth [5]. B cell development requires assembly of its antigen receptor loci through V(D)J recombination which occurs in developing B cells in fetal liver and bone marrow [6, 7]. Further development leads to migration to peripheral lymphoid organs such as the spleen where, upon antigen encounter, mature B cells can undergo another recombination process called class switch recombination (CSR). CSR enables IgM-expressing B cells to switch to the expression of other antibody classes, specified by different constant genes. Each constant gene is part of a transcription unit where transcription, termed germline (GL) transcription, initiates at an inducible promoter (called I promoter) and terminates downstream of the constant exons [8]. GL transcription is associated with various induced epigenetic changes (*e*.*g*. [9, 10]) and is controlled by different *cis*-regulatory elements including enhancers and insulators (*e*.*g*. [11–14]). In particular, the 3′ regulatory region (3′RR), which contains four enhancers located downstream of the *IgH* locus, effects a long-range enhancing activity on the multiple I promoters [15].

While V(D)J recombination targets all antigen receptor loci in B and T lymphocytes [7], CSR is strictly B-cell specific and targets exclusively the immunoglobulin heavy chain (*IgH*) locus [8]. This highly restricted targeting raises important developmental questions. For instance, it is still unknown whether all the epigenetic features of the *IgH* constant locus are acquired *de novo* in the B cell lineage and at the right B cell developmental stage, *i*.*e*. when GL transcription occurs, or whether the locus is at least in part epigenetically pre-marked.

Here, we focused on DNA methylation and used bisulphite sequencing to analyze the methylation profiles of multiple *cis*-acting elements at the *IgH* constant locus. We show that the methylation patterns of most *cis*-acting elements are established and faithfully maintained independently of B cell activation or GL transcription. Moreover, one I promoter, but not enhancers, was hypomethylated early during ontogeny and recruited different demethylation pathways.

## Results

### Induction of GL transcription and CpG demethylation in primary B cells

Splenic B cells can be activated by various extracellular signals (mitogen, cytokines…). Each stimulation condition induces a specific (set of) I promoter(s) and directs CSR to the corresponding constant gene(s) [8]. We checked induction of GL transcription and as expected, RT-qPCR and FACS revealed high levels of GL transcripts and robust CSR upon appropriate stimulation (**S1 Fig**).

To analyze methylation profiles of I promoters, we used bisulphite sequencing. Because this technique does not discriminate 5-methylcytosine from 5-hydroxymethylcytosine, a fraction of methylated cytosines may include 5-hydroxymethylcytosines. Conversely, a fraction of unmethylated cytosines may include 5-carboxylcytosines and 5-formylcytosines. Throughout this study, we did not quantify the levels of the oxidized methylcytosines.

**Fig 1:**
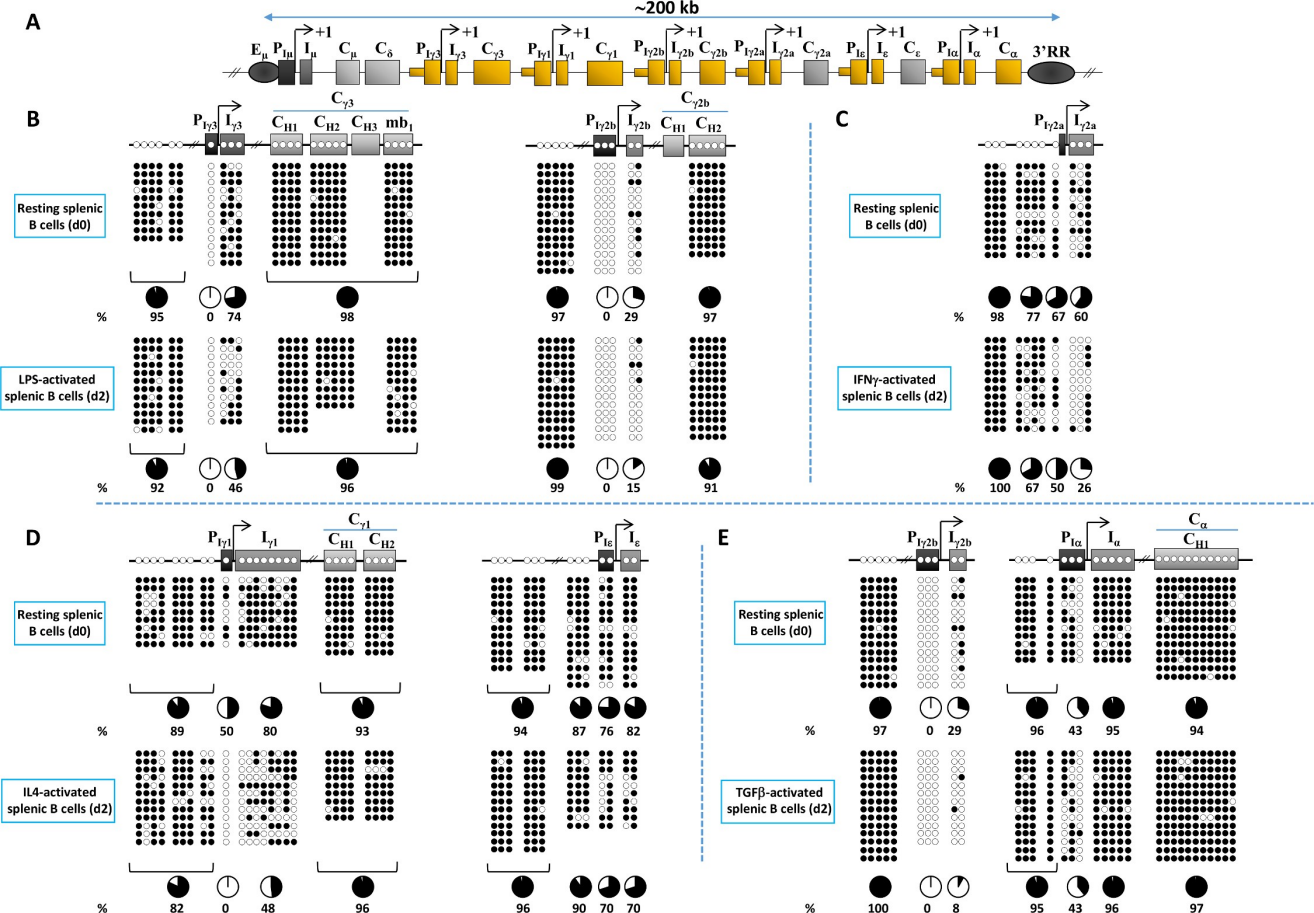
DNA methylation patterns at the *IgH* constant locus in resting and stimulated splenic B cells. **(A)** Scheme of the mouse *IgH* constant locus. The CpGs sequenced map to the regions depicted in orange. These include GL promoters (P_Ix_) and upstream regions, I exons (I_x_) and Cγ3, Cγ1, Cγ2b and Cα exons. Note that the distal CpG at Iγ2b promoter maps right upstream of the distal SMAD3 binding site (see S2 Fig), therefore, there is an uncertainty whether this CpG is truly part of the core promoter. The arrow indicates transcription start site. For convenience, only one initiation site is shown but GL transcripts initiate at multiple sites. Eμ enhancer acts as an enhancer and a GL promoter (hence Eμ/PIμ). The 3’RR is also indicated downstream of the locus (not to scale). **(B-E)** Genomic DNAs were purified from CD43^-^ WT splenic B cells (d0) and at day 2 (d2) post-stimulation and assayed by bisulphite sequencing. The top panels represent the sequencing results from resting splenic B cells (d0). The bottom panels represent those from activated splenic B cells (d2): **(B)** LPS stimulation (which induces Iγ3 and Iγ2b promoters), **(C)** IFNγ stimulation (induces Iγ2a promoter), **(D)** IL4 stimulation (induces Iγ1 and Iε promoters), **(E)** TGFβ stimulation (induces Iγ2b and Iα promoters). The localization of the sequenced CpGs is displayed in the upper schemes. The unmethylated and methylated cytosines are represented by open and filled circles, respectively. The percentage of methylation is indicated underneath the circles for the indicated elements. Note that the panel of Iγ2b region in resting B cells (d0) is the same in (B) and (E). Iγ2a promoter does not contain any CpG, the most proximal lies at 88 bp upstream of RUNX1 binding site. See S2 Fig and S2 Table for additional information on the localization of, and the distance between, the sequenced CpGs.

In order to determine if and how CpG methylation patterns are affected upon induction of GL transcription, we first compared the methylation state of all CpGs at I promoters and flanking sequences (**Fig 1A**), in resting and activated splenic B cells. We focused on the promoters’ CpGs to establish the link between DNA methylation and transcription initiation, but we also analyzed I exons and different constant exons as sites of transcriptional elongation. Analysis of some CpGs upstream of the promoters, located outside the transcription units and the known regulatory regions, served as “negative controls” as we anticipated them to be hypermethylated (**S2 Fig and S3 Fig)**.

Inspection of the data revealed various unexpected aspects of CpG methylation in the *IgH* constant locus. In particular: Most CpGs upstream of the promoters were heavily methylated in resting B cells and remained so after activation (**Fig 1B–1E and S3 Fig**). Strikingly, some promoters’ CpGs, notably the unique CpG at Iγ3 (see discussion), three CpGs at Iγ2b, and one CpG at Iα promoters, were fully unmethylated in resting B cells (**Fig 1B and 1E**). At the promoters, there was no obvious correlation between promoter activation and CpG demethylation (**Fig 1B–1E**), except for the Iγ1 promoter’s unique CpG, which lost all methylation upon IL4 activation (**Fig 1D**). The nature of the stimulus did not alter the CpG methylation state of Iγ2b promoter as a similar pattern was observed following either LPS or TGFβ stimulation, which both activate this promoter (**Fig 1B and 1E**). A positive correlation between induction of GL transcription and CpG demethylation could be seen for specific, mostly proximal, CpGs at Iγ3, Iγ1, Iγ2b and Iγ2a exons (hereafter Iγ exons). In contrast, the CpGs of Iε and (more markedly) Iα exons remained hypermethylated (**Fig 1B–1E**). CpG methylation status of all constant exons studied (Cγ3, Cγ1, Cγ2b, and Cα) was unchanged upon appropriate activation (**Fig 1B, 1D and 1E**). The targeting of CpGs for (de)methylation is highly focused, *i*.*e*., there is no evidence for spreading of this epigenetic mark as best illustrated by the hypomethylated CpG of Iα promoter (**Fig 1E and S3 Fig**) (see below).

### The demethylated state of Iγ3 and Iγ2b promoters is independent of their activity

**Fig 2:**
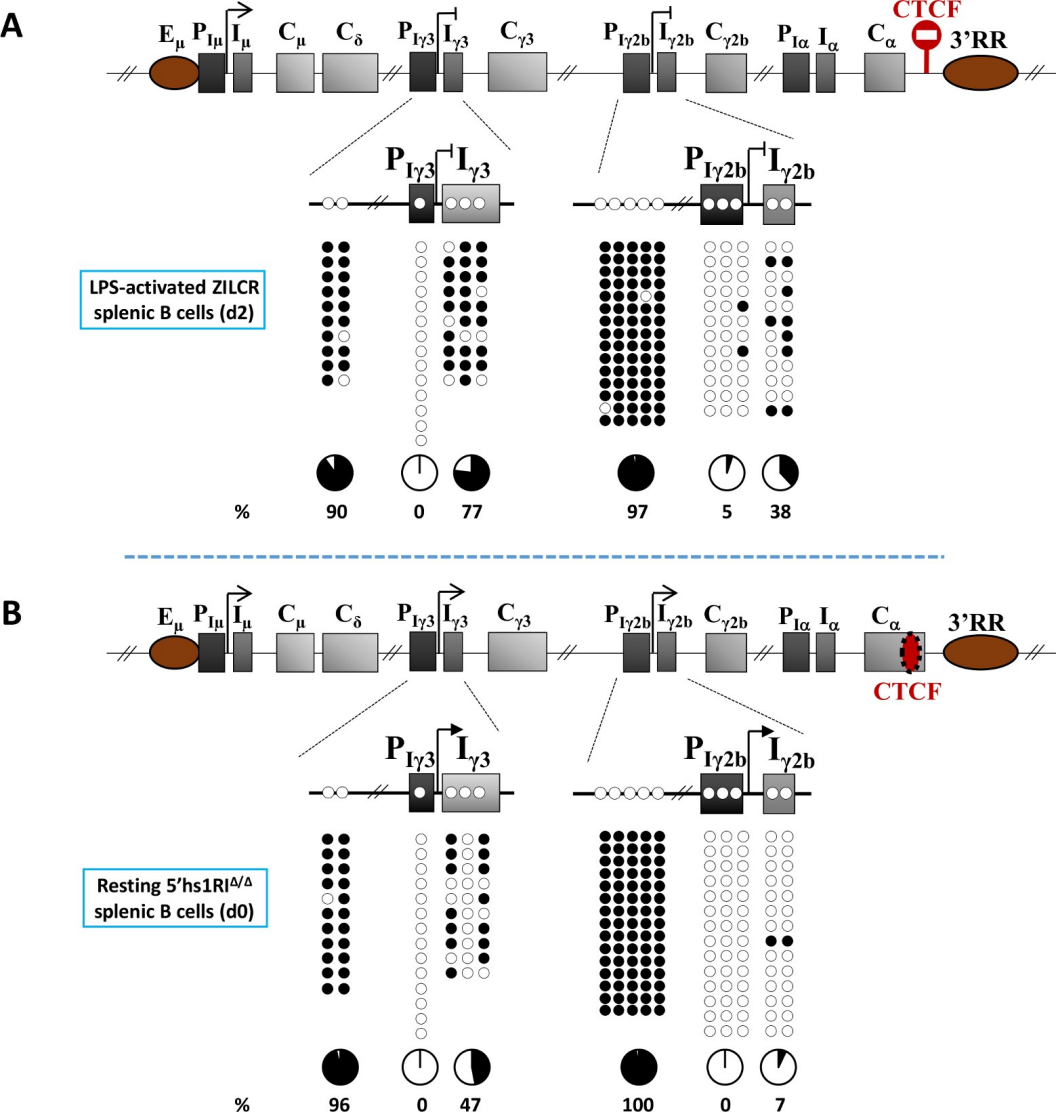
DNA methylation and transcriptional activity of Iγ3 and Iγ2b promoters. **(A)** The upper scheme indicates the insertion site of the ectopic CTCF insulator (in red) right upstream of the 3’RR (ZILCR mouse line). The insertion results in a complete shut-down of Iγ3 and Iγ2b promoters upon LPS stimulation. The panels report the results of bisulphite sequencing (d2 post-LPS stimulation). **(B)** The upper scheme indicates the deletion of the endogenous CTCF insulator within α constant gene (dotted red oval) (5’hs1RI mouse line). The deletion results in a constitutive activity of Iγ3 and Iγ2b promoters in resting B cells. The panels show the results of bisulphite sequencing on CD43^-^ sorted splenic B cells (d0).

In order to determine whether CpG demethylation occurs as a consequence of B cell activation or whether it is a direct consequence of GL transcription *per se*, we investigated CpG methylation in genetic contexts where Iγ3 and Iγ2b promoters were silenced in *activated* B cells, or constitutively active in *resting* B cells. In ZILCR mouse line, the chicken β-globin core insulator was inserted upstream of the 3’RR, resulting in a complete silencing of Iγ3 and Iγ2b promoters upon LPS stimulation (Braikia and Khamlichi, in preparation). In the second mouse model, the 5’hs1RI CTCF insulator within the Cα constant gene was deleted, leading to constitutive activity of Iγ3 and Iγ2b promoters in resting B cells [13] (**Fig 2A and 2B**).

The unmethylated state of Iγ3 and Iγ2b promoters remained essentially unchanged in LPS-activated ZILCR B cells (**Fig 2A**), and in unstimulated 5’hs1RI splenic B cells (**Fig 2B**). In LPS-activated ZILCR B cells, the methylation pattern of Iγ3 and Iγ2b exons was comparable to that seen in WT resting B cells (**Fig 1B and Fig 2A**). When Iγ3 and Iγ2b promoters were active in the absence of B cell activation, a lack of methylation was seen at exons Iγ3 and Iγ2b that was globally similar to that in LPS-activated WT B cells (**Fig 1B and Fig 2B**).

Taken together, the data from WT and mutant splenic B cells demonstrate that the unmethylated state of Iγ3 and Iγ2b promoters is locally established prior to B cell activation and transcription induction, and is maintained independently of B cell activation and promoter activity. Additionally, insulation of the 3’RR does not affect the methylation pattern of Iγ3 and Iγ2b promoters. In contrast, the relative demethylation of Iγ3 and Iγ2b exons results from GL transcription and not from B cell activation.

### Methylation patterns of *IgH cis*-acting elements are established and maintained independently of B cell activation

**Fig 3:**
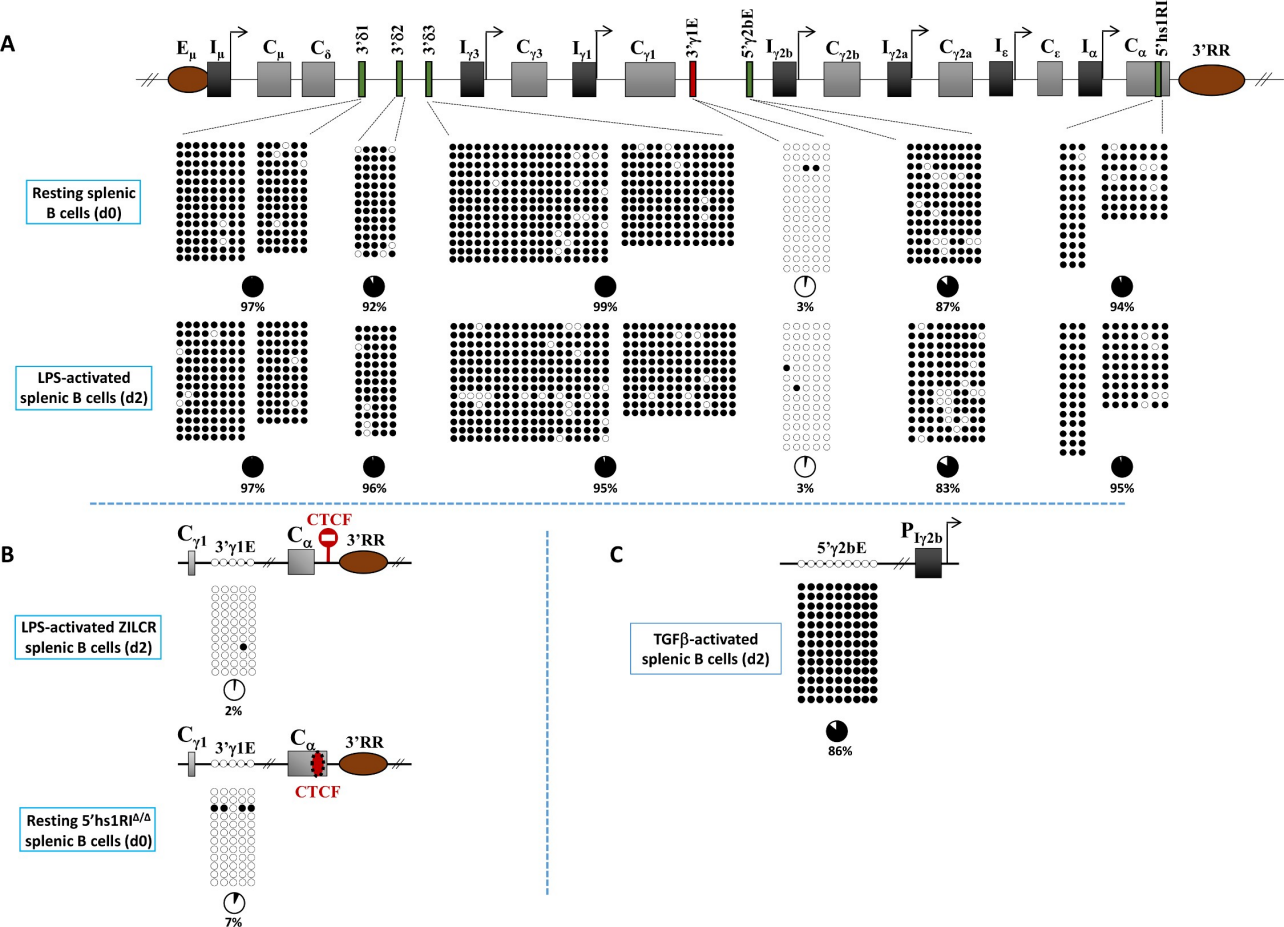
DNA methylation patterns of different *cis*-acting elements of the *IgH* constant locus in resting and activated splenic B cells. **(A)** Top. Scheme of the *IgH* constant locus depicting the relative position of the mapped elements, in red (3’γ1E) and in green (3’δ1, 3’δ2, 3’δ3, 5’γ2bE and 5’hs1RI) (see text for details). Bottom. Bisulphite sequencing assays were performed on genomic DNAs from purified WT resting splenic B cells (d0, top panels) and from activated splenic B cells (d2 post-LPS stimulation, bottom panels). **(B)** The hypomethylated state of the 3’γ1E is not affected by ectopic insulation of the 3’RR (top panel) or by deletion of the endogenous 5’hs1RI insulator. **(C)** The hypermethylated state of the 5’γ2bE does not vary upon TGFβ stimulation (compare with LPS stimulation in (A)). The 9 CpGs coincide with the DNaseI hypersensitive site (see text for details), and are right upstream of the 5 CpGs shown in Figs 1 and 2, which illustrates the hypermethylated state of the region upstream of Iγ2b (see S2 Fig and S2 Table for the localization of, and the distance between, the sequenced CpGs).

Iγ3 and Iγ2b promoters were unmethylated prior to, and following B cell activation, reminiscent of Eμ enhancer and the 3’RR [16–19]. This led us to explore the methylation pattern of other *cis*-acting elements, with known or suspected regulatory function. We focused on three CpG-rich clusters at Cδ-Iγ3 intergenic region (3’δ1 to 3’δ3) (**Fig 3A**). Two clusters (3’δ1 and 3’δ2) flank a region that is highly enriched in transcription factors binding sites and may play a role in early B cell development [20]; the other, located further downstream, is used as a negative control. We also examined two DNase I hypersensitive sites within Cγ1-Iγ2b intergenic region (hereafter 3’γ1E and 5’γ2bE) that bind various transcriptional/architectural factors [21, 22] and are involved in long-range interactions with multiple regulatory elements of the *IgH* locus in early B cells [21]. Additionally, 3’γ1E displays enhancer activity in pro-B cells [22]. Finally, we analyzed the intragenic 5’hs1RI insulator region whose CTCF binding site does not contain any CpG but is flanked by two clusters of 3 and 11 CpGs [13].

The data showed distinct CpG methylation patterns: 3’γ1E was largely unmethylated, both in resting and LPS-activated splenic B cells (**Fig 3A**) and its pattern was unchanged upon insulation of the 3’RR or deletion of 5’hs1RI (**Fig 3B**). The 3’δ1–3, 5’γ2bE and 5’hs1RI elements were hypermethylated in resting B cells as well as after LPS activation (**Fig 3A**). 5’γ2bE CpGs were also methylated in TGFβ-activated splenic B cells (**Fig 3C**).

### Ontogenic, lineage-, and cell-type specific CpG demethylation of Iγ3, Iγ2b promoters and 3’γ1E enhancer

The finding that Iγ3 and Iγ2b promoters and 3’γ1E enhancer were essentially unmethylated in resting splenic B cells led us to investigate when their non-methylated state was established, and whether this state was B cell-specific. To this end, we analyzed CpG methylation in various tissues and cell types. As controls, we assayed the Eμ enhancer, known to undergo lymphoid-specific demethylation and to remain unmethylated throughout B cell development [17, 19], and 5 CpGs upstream of Iγ2b which were heavily methylated in splenic B cells (**Figs 1 and 2**).

**Fig 4:**
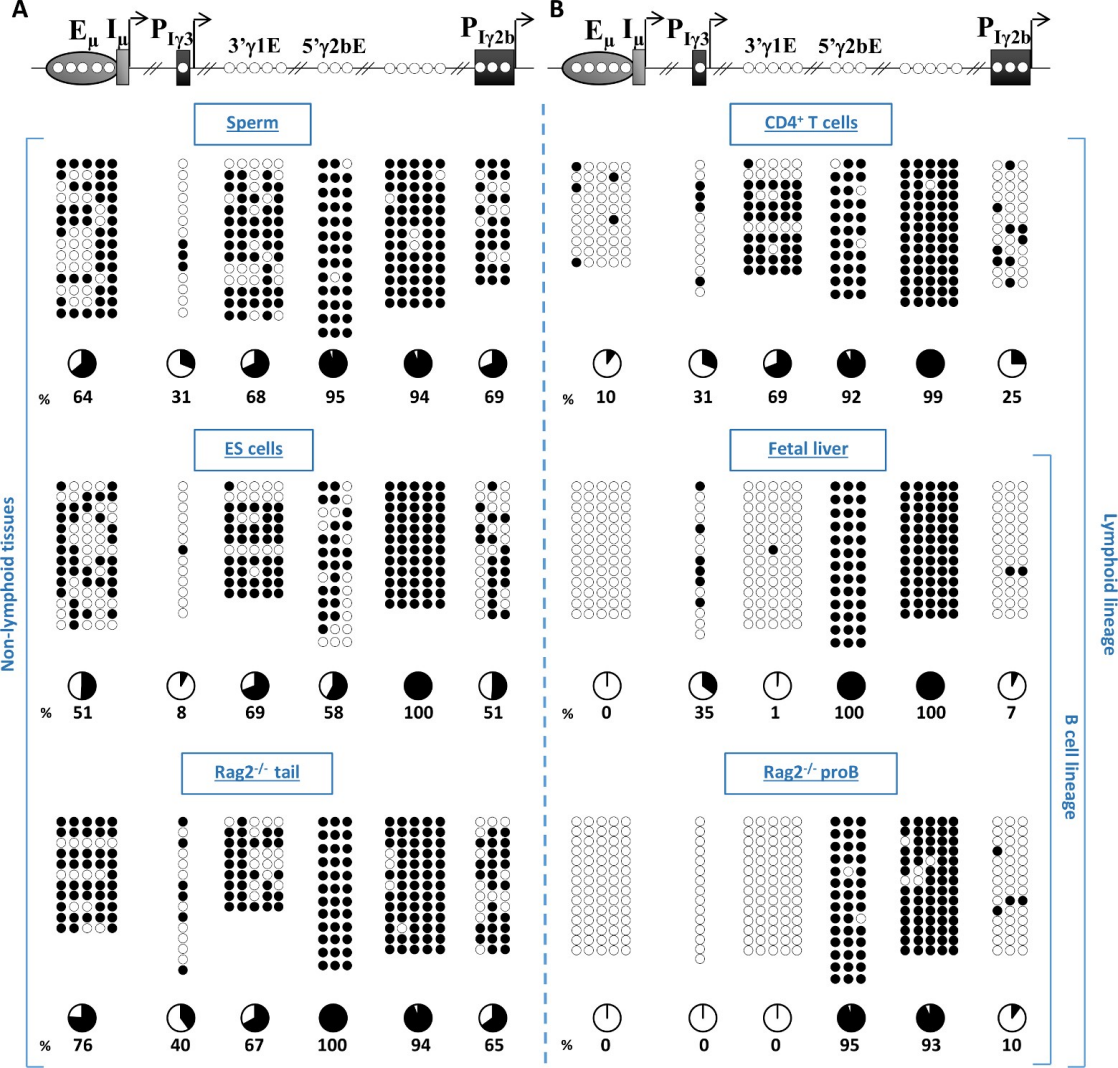
Methylation patterns of *IgH cis*-acting elements during ontogeny. The top scheme indicates the *cis*-acting elements mapped (Eμ/Iμ enhancer/GL promoter; Iγ3 and Iγ2b promoters, and 3’γ1E and 5’γ2bE). The 5 hypermethylated CpGs upstream of Iγ2b promoter (as determined in resting B cells, see Figs 1 and 2) were included as a “negative” control. For 5’γ2bE, only 3 CpGs (out of the 9 CpGs shown in Fig 3) were analyzed. See also the note on the distal CpG at Iγ2b promoter in the legend to Fig 1. Bisulphite sequencing maps for the indicated *cis*-acting elements were determined. **(A)** In non-lymphoid cells/tissues: mature sperm (top), ESCs (middle), tail of *Rag2*-deficient mice (devoid of any circulating lymphocytes) (bottom). **(B)** In lymphoid cells: splenic CD4^+^ T cells (top), fetal liver B cells (middle), and *Rag2*-deficient pro-B cells (bottom).

Indeed, The 5 CpGs upstream of Iγ2b were hypermethylated regardless of the cell type analyzed (**Fig 4A and 4B**). In contrast, Eμ was only minimally methylated in CD4^+^ T cells (10% of mCpGs), and was fully unmethylated in WT fetal liver B cells and in pro-B cells derived from the bone marrow of *Rag2*-deficient mice (**Fig 4B**). However, Eμ was relatively more methylated in mature sperm (64%), in serum-grown embryonic stem cells (ESCs) (51%) and in the tail tissue of *Rag2*-deficient mice (76%) (**Fig 4A**). The 5’γ2bE was heavily methylated in all tissues and cell types analyzed except in ESCs where it was relatively less methylated (58%) (**Fig 4A and 4B**). Interestingly, 3’γ1E underwent a strict B cell-specific demethylation, contrasting with Eμ enhancer whose demethylation was more pronounced in T cells (**Fig 4B**). Importantly, Iγ3 promoter was markedly hypomethylated in sperm (31%) (**Fig 4A**), whereas Iγ2b promoter (69%) (**Fig 4A**) and Iγ1 (100%) and Iα (81%) promoters (**S4 Fig**) were heavily methylated. Importantly, Iγ3 promoter and, to lesser extent, Iγ2b promoter underwent further demethylation in ESCs (8% and 51% respectively) (**Fig 4A**). In non-B cells, compared to ESCs, Iγ3 and Iγ2b were more methylated in *Rag2*^-/-^ tail (40% and 65% respectively) (**Fig 4A**), whereas in CD4^+^ T cells, Iγ3 underwent moderate methylation (31%) while Iγ2b was further demethylated (25%) (**Fig 4B**). Remarkably, in the B cell lineage, Iγ2b promoter was more demethylated than Iγ3 promoter in fetal liver (7% and 35% of mCpGs). Iγ3 promoter became fully unmethylated in pro-B cells of *Rag2*-deficient mice (**Fig 4B and S5 Fig**).

Altogether, the data revealed that, among the *cis*-acting elements analyzed, Iγ3 promoter was already hypomethylated in sperm and ESCs, and fully unmethylated in pro-B cells of adult mice. Iγ2b promoter, Eμ and 3’γ1E enhancers were hypermethylated in sperm but underwent massive demethylation in fetal liver B cells.

### Pathways that influence CpG methylation differentially affect Iγ3 and Iγ2b promoters

The above data showed that Iγ3 and Iγ2b promoters displayed different dynamic methylation patterns during embryonic development and cell differentiation, and that in sperm and ESCs, Iγ3 promoter was hypomethylated compared to Iγ2b. One possibility is that the two promoters are targeted by different demethylation machineries. In an attempt to identify the demethylation pathways involved, we assayed for CpG methylation at Iγ3 and Iγ2b promoters in mature sperm and resting splenic B cells of mice with Activation-induced cytidine deaminase (AID), Uracil DNA glycosylase (UNG), Ataxia telangiectasia mutated kinase (ATM), or the Tumor suppressor protein p53 deficiency (see discussion).

**Fig 5:**
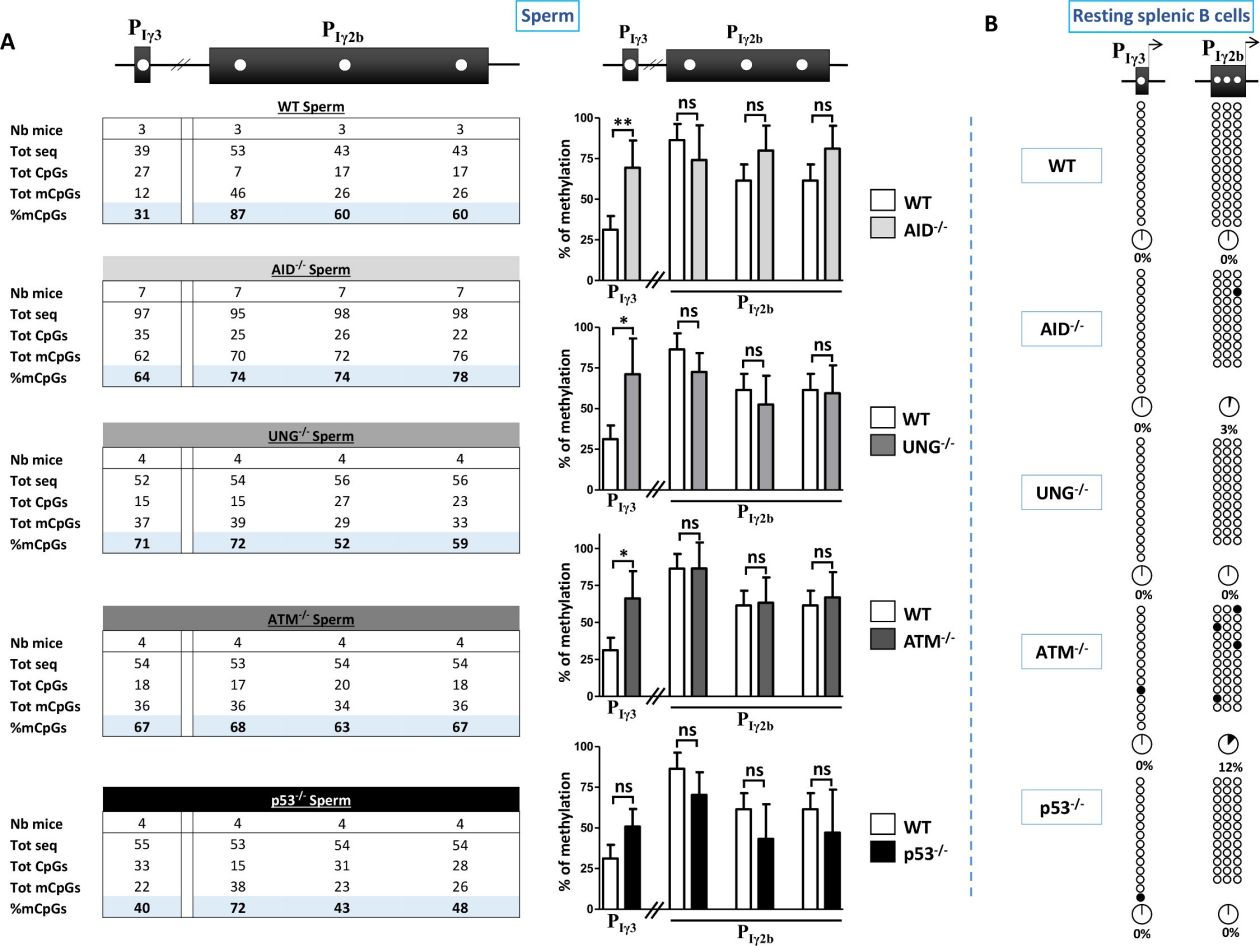
Pathways involved in the demethylation of Iγ3 promoter. **(A)** The table on the left summarizes the results of bisulphite sequencing of the CpGs analyzed (upper scheme) in sperm of WT adult males or with the indicated deficiencies. For each genotype, the number of mice, the total number of sequences, of demethylated CpGs (CpGs), of methylated CpGs (mCpGs), and the percentages of mCpGs (highlighted), are indicated. The histograms on the right show the statistical data for each individual CpG displayed on the upper scheme. The differences observed between WT and mutant mice were evaluated by unpaired t-test. The difference between means is significant if *p* < 0.05 (*), very significant if *p* < 0.01 (**); ns, not significant. **(B)** Methylation patterns of Iγ3 and Iγ2b promoters in resting splenic B cells with the indicated genotypes.

Strikingly, Iγ3 promoter displayed a hypermethylated pattern in AID-, UNG-, and ATM-deficient sperm compared to WT control (**Fig 5A**). In contrast, the methylation pattern of Iγ3 promoter did not significantly change in p53-deficient sperm (**Fig 5A**). The methylation pattern of Iγ2b promoter was not significantly affected regardless of the genetic deficiency (**Fig 5A**). For all deficiencies analyzed, the methylation pattern of Iγ3 and Iγ2b promoters was similar to WT in B cells (**Fig 5B**).

The data established that in mature sperm, Iγ3 and Iγ2b promoters displayed different methylation patterns, and that Iγ3 promoter was specifically hypermethylated in AID-, UNG-, and ATM-deficient sperm. In resting B cells however, the unmethylated profile of both promoters was essentially insensitive to AID, ATM, UNG, or p53 deficiency.

## Discussion

Four main conclusions emerge from this study. DNA methylation does not play a significant role in *IgH* constant genes expression. Acquisition of DNA methylation by the constant exons is not mediated by transcriptional elongation. The hypomethylated pattern of the late B cell-specific Iγ3 promoter was manifest in mature sperm and ESCs already, in contrast to Eμ and 3’γ1E enhancers and other I promoters. Iγ3 and Iγ2b promoters recruited different demethylation pathways.

Except for Iγ1, B cell activation and induction of GL transcription did not perturb the methylation patterns of I promoters. Explanations such as the nature of the stimulus or the number of promoter CpGs cannot explain these patterns. For instance, Iγ1 and Iε promoters are both induced by IL4 stimulation, but while Iγ1 underwent full demethylation, Iε did not. On the other hand, both Iγ3 and Iγ1 promoters contain a single CpG, but while Iγ3 was already unmethylated in resting B cells, Iγ1 became fully unmethylated only after induction. In this regard, various studies showed that methylation of a single CpG can have important functional or pathological consequences [23–26].

GL transcription at the *IgH* constant locus is largely controlled by the 3’RR [15], which was shown to engage in long-range interactions with I promoters through chromatin looping, in a stimulus-dependent manner [27–30]. Additionally, the 3’RR controls various active histone modifications at I promoter/exon regions [31]. Our findings strongly suggest that the formation of *IgH* loops and the set-up of active histone marks associated with I promoters activation do neither require nor induce demethylation of I promoters. Significantly, Iγ3 and Iγ2b promoters are the most sensitive to 3’RR mutations (*e*.*g*. [11, 12]). Nonetheless, their unmethylated pattern did not change upon insulation of the 3’RR, which fully repressed these promoters. The 3’RR thus controls Iγ3 and Iγ2b promoters through mechanisms that do not involve DNA methylation, contrasting in this regard with other *Ig* enhancers (*e*.*g*. [17, 19, 32]). It remains to be established whether the 3’RR displays a demethylating activity at earlier B cell developmental stages. Paradoxically, Iγ1, known to be relatively 3’RR-independent (*e*.*g*. [11, 12]), was the only I promoter whose demethylation was induced. This may relate to the presence of specific regulatory elements with demethylating activity such as the putative Iγ1 promoter-associated enhancer [33], and/or the 3’γ1E enhancer. Testing these hypotheses still awaits appropriate knock-out models.

Induction of GL transcription led to a moderate hypomethylation of essentially the most proximal CpGs of Iγ exons. This may be due to pausing of RNA pol II that takes place 30–60 nucleotides downstream of the transcription start site(s). Accordingly, high-levels of RNA pol II p-Ser5 were detected at Iγ3 exon upon LPS stimulation [9], which may protect some CpGs against methylation. Methylation of Iε and Iα exons, however, was not impacted by stimulation, suggesting that the mechanisms that underlie pausing at I exons may differ.

Seminal studies using transformed cell lines and methylation-sensitive restriction enzymes found a positive correlation between DNA hypomethylation and constant genes transcription [34–37]. However, this correlation was not observed in primary B cells [38]. Accordingly, we found that Cγ3, Cγ1, Cγ2b and Cα exons were already hypermethylated in unstimulated splenic B cells and remained so after induction of GL transcription, regardless of the nature of the stimulus. This indicates that transcriptional elongation across the chromatin of constant exons does not bring about any obvious change of their hypermethylated pattern. Interestingly, this hypermethylated pattern coincides with transcription-associated deposition of H3K36me3 at Cγ exons [9]. In genomic imprinting for instance, acquisition of DNA methylation through transcription-associated H3K36me3 has been demonstrated for some imprinting control regions. In this process, H3K4 methylation, which prevents the action of DNMT3A-DNMT3L *de novo* methyl-transferase complex, is first removed from chromatin, this enables transcription-associated H3K36me3 to recruit DNMT3A-DNMT3L complex that will methylate DNA [39]. This is clearly not the case for *IgH* constant exons which are likely methylated through a different mechanism.

Previous work indicated that H3K36me3 and intragenic DNA methylation contribute to the silencing of alternative, intragenic promoters [40, 41]. Low levels of antisense switch transcripts have been detected (*e*.*g*. [42, 43]), but antisense promoters have not been precisely defined. Intragenic methylation may contribute to down-regulation of the antisense promoters and/or other cryptic promoters. An attractive possibility could be that DNA hypermethylation and H3K36me3 across the constant exons protect these regions from AID attack by favoring a compacted chromatin structure after nucleosome displacement induced by RNA pol II passage. This chromatin-based protection mechanism is physiologically relevant as the constant exons are coding sequences whose reading frame must be preserved if the Ig heavy chain is to be produced. In this regard, the highly cytosine-rich, non-coding switch sequences, which are preferentially targeted by AID during CSR are strikingly poor in CpG [44] compared to constant exons.

Two major waves of DNA methylation reprogramming occur during development, shortly after fertilization and in primordial germ cells (PGCs). After the massive methylation erasure in PGCs, *de novo* DNA methylation is acquired in prenatal prospermatogonia before birth. The methylation patterns are fully established at birth and are maintained before the cells enter meiosis [45, 46], and it was shown that sperm cells display the highest global DNA methylation level [47]. In stark contrast to I promoters, and to Eμ and 3’γ1E enhancers, Iγ3 was already hypomethylated in mature sperm. Moreover, Iγ3 promoter underwent further demethylation in serum-grown ESCs despite the fact that ESCs grown in this condition display high DNA methylation levels [48], comparable to those of mature sperm [47]. These findings suggest that Iγ3 promoter is hypomethylated in pre-implanted embryo. Upon differentiation however, Iγ3 promoter moderately acquires DNA methylation and is fully unmethylated only in B cells of adult mice. Altogether, the above findings strongly suggest that the *cis*-acting elements analyzed are targeted by (de)methylating activities in a highly specific manner. The differential targeting is also evident from the patterns of Iγ3 and Iγ2b in sperm with AID, UNG, ATM or p53 deficiency.

The role of AID in DNA demethylation is still controversial (*e*.*g*. [45, 46, 49–51]). AID was implicated both *in vitro* and *in vivo* at various stages of mouse embryonic development [47, 52, 53]. Our data show that Iγ3, but not Iγ2b, is hypermethylated in AID-deficient sperm. This indicates that AID demethylation pathway is involved, and preferentially targets Iγ3 promoter. Whether it is AID itself, or a cofactor, that is directly implicated is presently unclear. Also, we do not infer that AID-mediated demethylation occurs in mature sperm. Demethylation may have occurred in PGCs, and the hypomethylated pattern subsequently maintained during the establishment of the male germ line. In this regard, low levels of AID expression were detected in PGCs but not in the germ line [52, 54].

Preferential targeting of Iγ3 promoter was also evident in UNG-deficient sperm. The base excision repair pathway [54], and in particular UNG which excises uracil from DNA, has been implicated in DNA demethylation in zygotes and PGCs [53, 55, 56]. In antibody diversification mechanisms in B cells, AID deaminates a non-methylated cytosine to generate a U:G mismatch that can be processed by UNG [57]. A somewhat analogous scenario has been proposed for cytosine demethylation in mouse zygotes [55]. Whether, similarly, UNG acts downstream of AID in PGCs is presently unclear. However, it is possible that UNG is involved through an AID-independent pathway.

ATM is a major component of the DNA damage response, and it has recently been implicated in the establishment of DNA methylation patterns during spermatogenesis, as global DNA methylation was reduced in ATM-deficient testis [58]. Based on this, we expected a hypomethylated pattern in ATM-deficient sperm. However, Iγ3 was hypermethylated while Iγ2b was unaffected. This suggests that ATM-mediated demethylation of Iγ3 implicates different, yet unknown mechanisms. In contrast, methylation patterns of Iγ3 and Iγ2b promoters did not significantly change in p53-deficient sperm. p53 has been shown to down-regulate the *de novo* DNMT3A and DNMT3B methyl-transferases and up-regulate TET1 and TET2 in naïve ESCs, whereas in differentiated cells, p53 became a repressor of *Tet1* and *Tet2* genes [59]. None of these modes of regulation seems to target Iγ3 and Iγ2b promoters although an effect of p53 at a discrete developmental stage can not be excluded.

Overall, the methylation pattern of Iγ3, but not of Iγ2b promoter, was perturbed in AID, UNG or ATM-deficient sperm. This suggests that different pathways somehow contribute to the setting of the methylation patterns of these promoters. Whether these pathways act at the same developmental stage and whether they interact with each other and/or with other pathways is presently unknown. In contrast, none of the pathways studied was significantly required for the maintenance of the unmethylated state of Iγ3 and Iγ2b promoters in resting splenic B cells. Thus, once the demethylation mark has been set up, the involved pathways seem dispensable for the maintenance of the mark at subsequent B cell developmental stages.

Though still debatable, accumulated evidence supports the notion that at least some of the epigenetic features that underlie tissue-specific expression are somehow stamped at earlier developmental stages, prior to the specification of the relevant lineage [39, 60, 61]. For instance, asynchronous replication, set up early during development, was suggested to epigenetically mark antigen receptor loci for mono-allelic recombination at the right developmental stage [62, 63]. Some, but not all, B cell-specific enhancers are primed in hematopoietic stem cells (*e*.*g*. [64–67]). Other tissue-specific genes are epigenetically marked in ESCs [68, 69]. Regarding DNA methylation specifically, different tissue-specific enhancers, but not promoters, displayed a subset of hypomethylated CpGs in ESCs [25, 70].

What could be the functional significance of the overall hypomethylated pattern of Iγ3 promoter? Splenic marginal zone B cells represent a special population of the adaptive immune system. These “innate-like” lymphocytes [71] play an important role in rapid protective responses against blood-borne antigens. They are in a state of active readiness and switch to IgG3 preferentially in response to T-cell-independent antigens [71]. We speculate that the early set-up of the hypomethylated pattern of Iγ3 may be part of an epigenetic programme that predisposes this promoter for fast activation in marginal zone B cells.

In conclusion, methylation patterns of *IgH* constant locus elements are essentially transcription-independent. The mature B cell-specific Iγ3 promoter is hypomethylated early during ontogeny. Iγ3 and Iγ2b promoters recruit different demethylation pathways that are dispensable for the maintenance of the demethylation mark once established in the B cell lineage. Further investigations are required to unravel the multiple facets of DNA methylation regulation at the *IgH* locus during development and to elucidate the mechanisms that control the process.

## Materials and methods

### Ethics statement

The experiments on mice were carried out according to the CNRS Ethical guidelines and were approved by the Regional Ethical Committee (Accreditation N° E31555005).

### ES cells, mice

ESCs (CK35 line, of 129Sv background) were provided by Chantal Cress (Institut Pasteur, Paris, France). The WT and homozygous *Rag2*^-/-^, ZILCR, 5’hs1RI^Δ/Δ^ were of 129Sv genetic background. AID^-/-^, ATM^-/-^, UNG^-/-^, and p53^-/-^ mutant mice were enriched in 129Sv genetic background through at least 8 back-crosses, and both their chromosomes 12 (harbouring the *IgH* locus) were derived from 129Sv. All the mice used were 6–8 week-old. ATM-deficient mice were purchased from Jackson labs and p53-deficient mice were from the European Mutant Mouse Archives, Orléans, France. AID-deficient mice were provided by T. Honjo, through C-A. Reynaud and J-C. Weill. UNG-deficient mice were provided by T. Lindahl, through C. Rada and the late M.S. Neuberger.

### Cell purification

Single cell suspensions from the bone marrows or spleens were obtained by standard techniques. *Rag2*-deficient pro-B cells (from the bone marrow of Rag2^-/-^ mice) and WT fetal liver B cells (at day 14 *post-coitum*) were positively sorted by using B220- and CD19-magnetic microbeads and MS columns (Miltenyi). Splenic B cells were negatively sorted by using CD43-magnetic microbeads and LS columns (Miltenyi). Splenic CD4^+^ cells were sorted as B220^-^IgM^-^CD4^+^ population. ESCs cells were serum-grown in the presence of LIF (10^6^ units/ml) throughout: first on mitomycin-treated feeder cells for 2 days, trypsinized and amplified for additional 2 days without feeders. After trypsinization, the cells were plated on gelatinized dishes for 2 hours, and the ESC-enriched supernatant carefully pipetted off and plated again for additional 2 hours in order to get rid of contaminating feeders. Sperm was collected from the cauda epididymis of adult males by the “swim-up” method [72].

### Induction of GL transcription

To induce GL transcription, negatively sorted CD43^-^ splenic B cells were cultured for 2 days, at a density of 5 x 10^5^ cells per ml in the presence of LPS (25 μg/ml) + anti-IgD-dextran (3 ng/ml) (hereafter LPS stimulation), LPS (25 μg/ml) + anti-IgD-dextran (3 ng/ml) + IL4 (25 ng/ml) (IL4 stimulation), LPS (25 μg/ml) + anti-IgD-dextran (3 ng/ml) + IFNγ (20 ng/ml) (IFNγ stimulation) or LPS (25 μg/ml) + anti-IgD-dextran (3 ng/ml) + IL4 (10 ng/ml) + IL5 (5 ng/ml) + BLyS (5 ng/ml) + TGFβ (2 ng/ml) (TGFβ stimulation).

### Genomic DNAs

Genomic DNAs were purified from the following sources: sorted resting splenic B cells from WT, 5’hs1RI^Δ/Δ^, AID^-/-^, UNG^-/-^, ATM^-/-^, and p53^-/-^ mutant mice; from WT, ZILCR, AID^-/-^ splenic B cells at day 2 post-stimulation; from WT ESCs, resting splenic CD4^+^ T cells, and fetal liver B220^+^ cells; from pro-B cells or from the tail of Rag2^-/-^ mice; from mature sperm of WT, UNG^-/-^, AID^-/-^, ATM^-/-^, and p53^-/-^ mutant mice.

### DNA methylation analyses

Purified genomic DNAs were assayed by sodium bisulphite sequencing by using a bisulphite conversion kit (Diagenode). Modified templates were amplified by PCR using converted primers listed in S1 Table. Converted primers were designed by using the public MethPrimer software. PCR products were separated by agarose gel electrophoresis, purified using QIAquick gel extraction kit (Qiagen), and cloned into pCR2.1-TOPO vector (Invitrogen). Transformed bacteria were plated immediately after transformation without pre-culture, and randomly picked clones were sequenced (Eurofins Genomics). Sequence analysis showed 99%-100% bisulphite modification efficiency.

### Antibodies and cytokines

Allophycocyanin (APC)-conjugated anti-B220, fluorescein isothiocyanate (FITC)-conjugated anti-IgG1, Phycoerythrin (PE)-conjugated anti-IgG2b, PE-conjugated anti-IgG2a, and PE-conjugated anti-CD4 antibodies were purchased from BioLegend. FITC-conjugated anti-IgG3 and FITC-conjugated anti-IgA were from BD-Pharmingen. LPS was purchased from Sigma, anti-IgD-dextran from Fina Biosolutions, TGFβ, B-LyS, IFNγ and IL5 from R&D, and IL4 from eBiosciences.

### Flow cytometry analyses (FACS)

At day 4 post-stimulation, B cells were washed and stained with anti-B220-APC and either anti-IgG3-FITC, anti-IgG2b-PE, anti-IgG1-FITC, anti-IgG2a or anti-IgA-FITC. Activated B cells from AID-deficient mice (unable to initiate CSR) were included throughout as negative controls. Data were obtained on 5 x 10^5^ viable cells by using a BD FACSCalibur flow cytometer.

### Reverse transcription-quantitative PCR (RT-qPCR)

Total RNAs were prepared from B cells at day 2 post-stimulation, reverse transcribed (Invitrogen) and subjected to qPCR using Sso Fast Eva Green (BioRad). Actin transcripts were used for normalization. The primers used have been described [13].

### Statistical analysis

Results are expressed as mean ± SD (GraphPad Prism) and overall differences between values from day 0 and day 2 post-stimulation were evaluated by paired t-test, and from WT and AID-, UNG-, ATM- and p53-deficient sperm by unpaired t-test. The difference between means is significant if *p* value < 0.05 (*), very significant if *p* value < 0.01 (**), and extremely significant if *p* value < 0.001 (***).

## Supporting information

S1 Fig Induction of GL transcription and class switching. CD43^−^ sorted splenic B cells from WT mice were induced to switch to IgG3 and IgG2b (LPS stimulation), to IgG2a (IFNγ stimulation), to IgG1 and IgE (IL4 stimulation) or to Ig2b and IgA (TGFβ stimulation). Total RNAs were prepared at day 0 and day 2 post-stimulation, reverse transcribed and the indicated spliced GL transcript levels were quantified by RT-qPCR (n≥4). *** *p*<0.001; ** *p*<0.01; * *p*<0.05. The scheme on the right shows the relative localization of the primers used to amplify the spliced forms of GL transcripts. GL transcripts initiate at multiple transcription start sites (for convenience, only one is indicated by the arrow), run across the S sequences and undergo polyadenylation downstream of the constant (C_x_) exons. Splicing enables fusion of I exon to the C exons and excision of the intervening sequences. Note that γ2a and α primary GL transcripts have three splice donor sites, the primers used amplify only one form of spliced transcript. At day 4 post-stimulation, the cells were stained with the indicated antibodies. Activated AID-deficient B cells are unable to switch and are included as negative controls. Representative plots are shown (n≥3). There are currently no reliable antibodies to stain surface IgE, therefore, we did not perform the corresponding FACS.
(TIF)

S2 Fig Localization of the sequenced CpGs with regard to known transcription factors binding sites and relative distances between them. The schemes represent the relative position of the mapped CpGs at the promoters and upstream regions, I exons, Cγ3, Cγ1, Cγ2b and Cα constant exons, and Cγ1-Iγ2b intergenic region. While all promoters CpGs are conserved between 129Sv and C57BL/6 mouse strains, there are differences between few CpGs outside the promoters. Orange circles indicate discrete CpGs for which we could not design reliable converted primers, or that are not conserved between 129Sv and C57/Black6 mouse lines, or that we did not target in this study. For the sake of clarity, not all these CpGs are shown. Where it applies, their number is indicated below (*e*.*g*. there are 12 CpGs between C_H3_ and mb1 exons of Cγ3 gene, hence x12). The numbers in red indicate the length of the sequence (in bp) encompassing the indicated CpGs, bordered by the first and the last CpGs (*e*.*g*. the 8 CpGs at Iγ1 exon are contained within 384 bp, which is also the distance between the first and the last CpGs). The numbers in blue indicate the distance (in bp) between the sequenced CpGs. I promoters, 3’γ1E and 5’γ2bE are magnified in the upper schemes to indicate the position of the CpGs relative to transcription factor (TF) binding sites and TSSs (indicated by a single arrow). The data on TFs as well as on the multiple TSSs are compiled from [21, 22, 33], from supplementary references [1–20] in S1 Text, and from bioinformatics analyses using the JASPAR database.
(TIF)

S3 Fig Percentages of methylated CpGs at GL promoter/exon regions in resting and activated splenic B cells. Genomic DNAs were purified from resting (d0) and activated (d2) splenic B cells, and assayed by bisulphite sequencing. The indicated d2 correspond to **(A)** LPS stimulation, **(B)** IL4 stimulation, **(C)** IFNγ stimulation, **(D)** TGFβ stimulation. For the sake of simplicity, only the most proximal hypermethylated CpGs upstream of I promoters are shown in the upper schemes (see Fig 1).
(TIF)

S4 Fig Iγ1 and Iα GL promoters are hypermethylated in the sperm of WT adult males. Bisulphite sequencing maps of the CpGs at Iγ1 and Iα GL promoters are shown (compare to Fig 4A and Fig 5A).
(TIF)

S5 Fig Methylation pattern of Iγ3 and Iγ2b promoters during ontogeny. The scheme recapitulates the methylation states of Iγ3 (red) and Iγ2b (yellow) promoters at various stages of development. For convenience, among non-B cells, only sperm and ESCs are shown.
(TIF)

S1 Text References associated with S2 Fig. (DOCX)

S1 Table PCR oligonucleotides used in this study. (PDF)

S2 Table Localization of the converted primers within the sequence of the mouse *IgH* locus (129S1 strain. GenBank accession number: AJ851868.3).
(PDF)
