# Supplementary figures and images for: Developmental regulation of DNA cytosine methylation at the immunoglobulin heavy chain constant locus
Source: PLoS Genet. 2019 Feb 19;15(2):e1007930. doi: 10.1371/journal.pgen.1007930 (PMC6380546; doi:10.1371/journal.pgen.1007930)

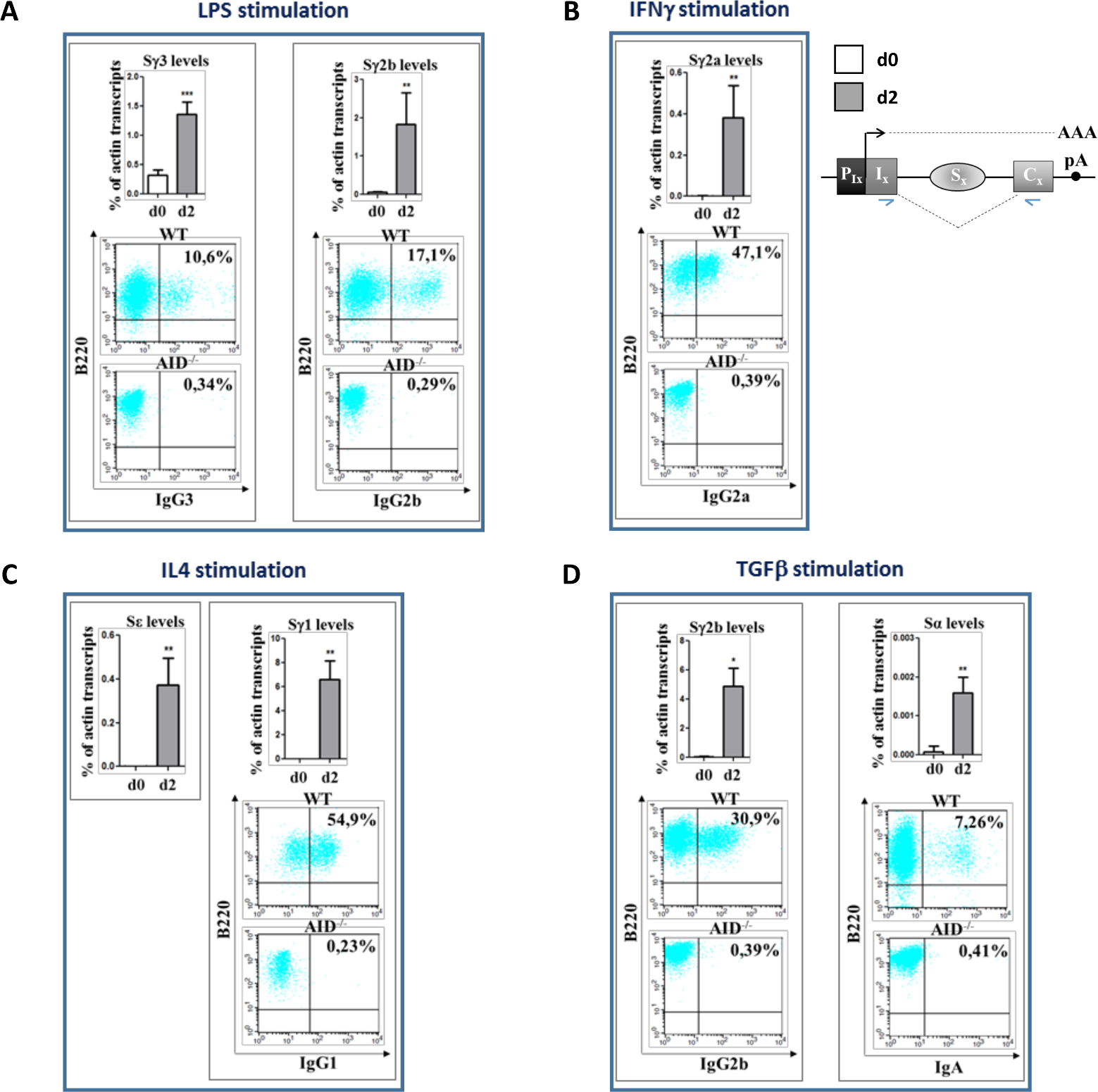

Supplement: S1 Fig — CD43− sorted splenic B cells from WT mice were induced to switch to IgG3 and IgG2b (LPS stimulation), to IgG2a (IFNγ stimulation), to IgG1 and IgE (IL4 stimulation) or to Ig2b and IgA (TGFβ stimulation). Total RNAs were prepared at day 0 and day 2 post-stimulation, reverse transcribed and the indicated spliced GL transcript levels were quantified by RT-qPCR (n≥4). *** p<0.001; ** p<0.01; * p<0.05. The scheme on the right shows the relative localization of the primers used to amplify the spliced forms of GL transcripts. GL transcripts initiate at multiple transcription start sites (for convenience, only one is indicated by the arrow), run across the S sequences and undergo polyadenylation downstream of the constant (Cx) exons. Splicing enables fusion of I exon to the C exons and excision of the intervening sequences. Note that γ2a and α primary GL transcripts have three splice donor sites, the primers used amplify only one form of spliced transcript. At day 4 post-stimulation, the cells were stained with the indicated antibodies. Activated AID-deficient B cells are unable to switch and are included as negative controls. Representative plots are shown (n≥3). There are currently no reliable antibodies to stain surface IgE, therefore, we did not perform the corresponding FACS. (TIF) [file pgen.1007930.s001.tif]

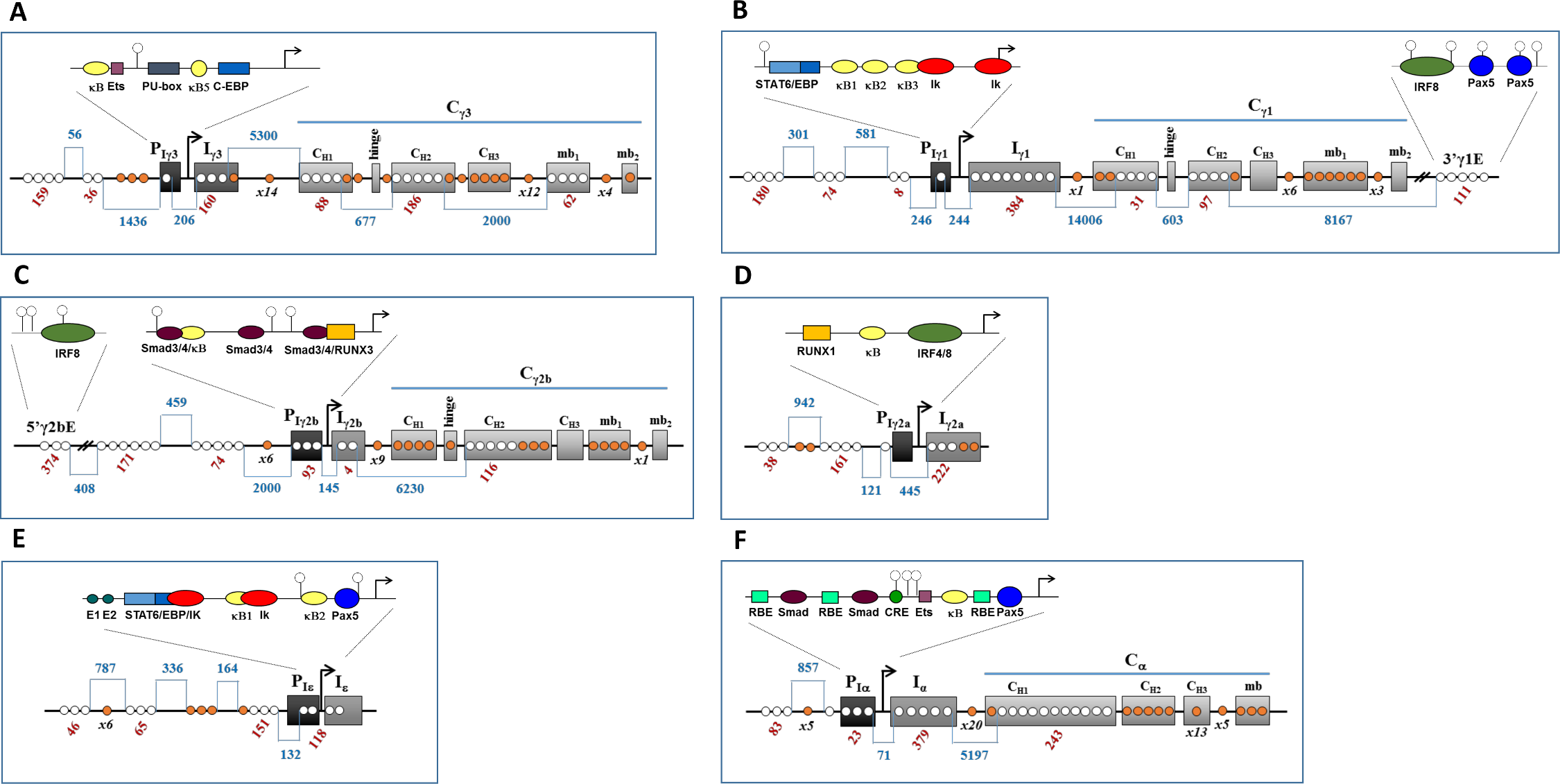

Supplement: S2 Fig — The schemes represent the relative position of the mapped CpGs at the promoters and upstream regions, I exons, Cγ3, Cγ1, Cγ2b and Cα constant exons, and Cγ1-Iγ2b intergenic region. While all promoters CpGs are conserved between 129Sv and C57BL/6 mouse strains, there are differences between few CpGs outside the promoters. Orange circles indicate discrete CpGs for which we could not design reliable converted primers, or that are not conserved between 129Sv and C57/Black6 mouse lines, or that we did not target in this study. For the sake of clarity, not all these CpGs are shown. Where it applies, their number is indicated below (e.g. there are 12 CpGs between CH3 and mb1 exons of Cγ3 gene, hence x12). The numbers in red indicate the length of the sequence (in bp) encompassing the indicated CpGs, bordered by the first and the last CpGs (e.g. the 8 CpGs at Iγ1 exon are contained within 384 bp, which is also the distance between the first and the last CpGs). The numbers in blue indicate the distance (in bp) between the sequenced CpGs. I promoters, 3’γ1E and 5’γ2bE are magnified in the upper schemes to indicate the position of the CpGs relative to transcription factor (TF) binding sites and TSSs (indicated by a single arrow). The data on TFs as well as on the multiple TSSs are compiled from [21, 22, 33], from supplementary references [1–20] in S1 Text, and from bioinformatics analyses using the JASPAR database. (TIF) [file pgen.1007930.s002.tif]

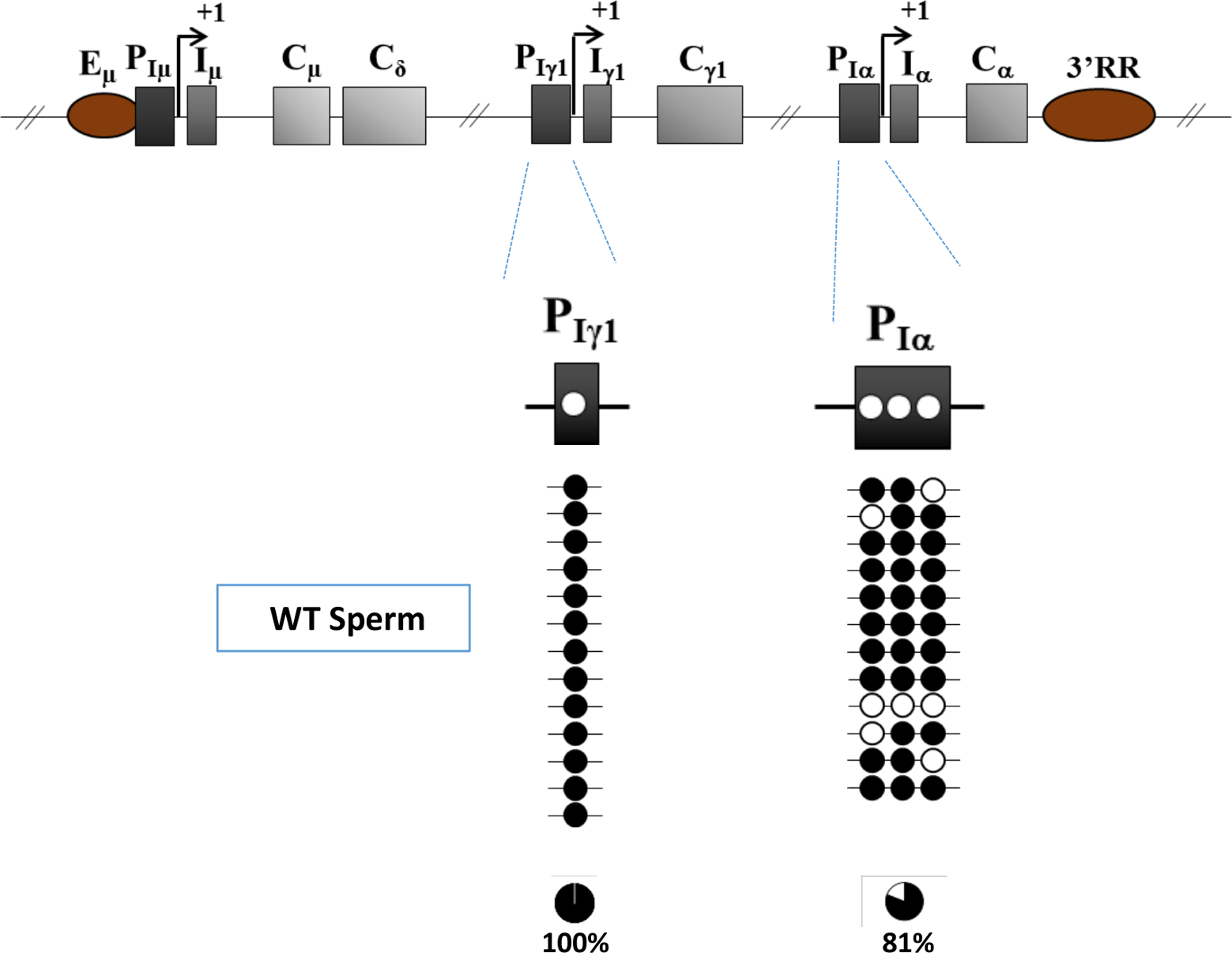

Supplement: S3 Fig — Genomic DNAs were purified from resting (d0) and activated (d2) splenic B cells, and assayed by bisulphite sequencing. The indicated d2 correspond to (A) LPS stimulation, (B) IL4 stimulation, (C) IFNγ stimulation, (D) TGFβ stimulation. For the sake of simplicity, only the most proximal hypermethylated CpGs upstream of I promoters are shown in the upper schemes (see Fig 1). (TIF) [file pgen.1007930.s003.tif]

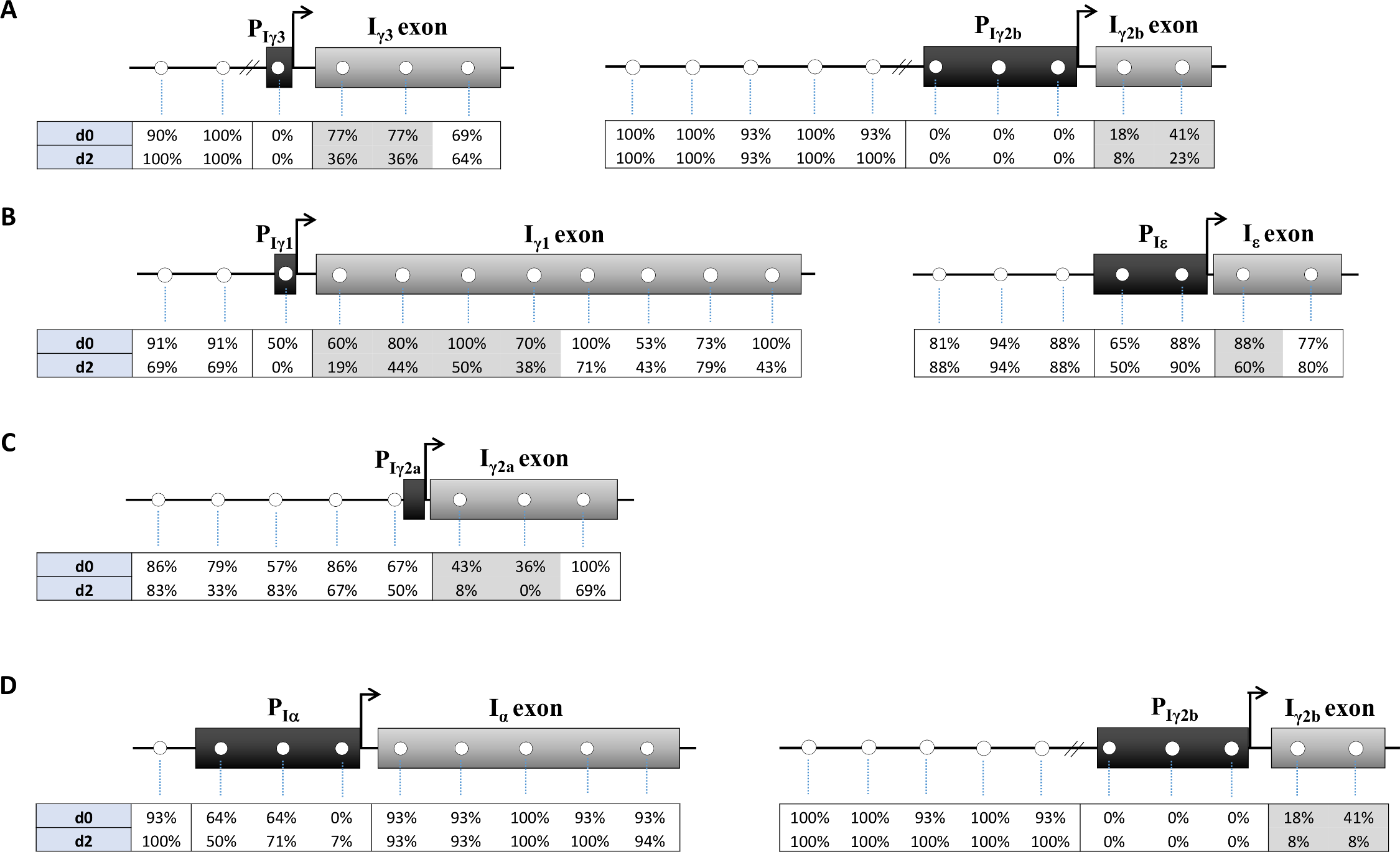

Supplement: S4 Fig — Bisulphite sequencing maps of the CpGs at Iγ1 and Iα GL promoters are shown (compare to Fig 4A and Fig 5A). (TIF) [file pgen.1007930.s004.tif]

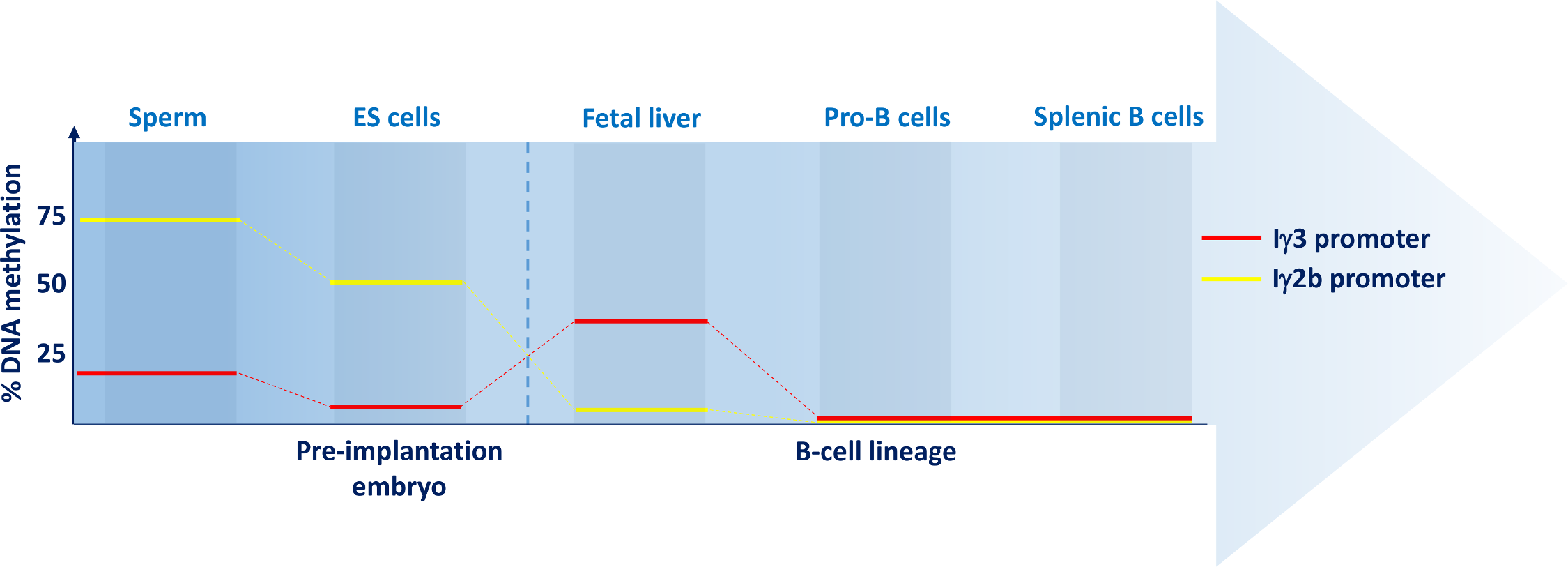

Supplement: S5 Fig — The scheme recapitulates the methylation states of Iγ3 (red) and Iγ2b (yellow) promoters at various stages of development. For convenience, among non-B cells, only sperm and ESCs are shown. (TIF) [file pgen.1007930.s005.tif]
