# Supplementary material for: Developmental regulation of DNA cytosine methylation at the immunoglobulin heavy chain constant locus
Source: PLoS Genet. 2019 Feb 19;15(2):e1007930. doi: 10.1371/journal.pgen.1007930 (PMC6380546; doi:10.1371/journal.pgen.1007930)
Supplement: S1 Text — (DOCX) [file pgen.1007930.s006.docx]

**S1 Text**

**Supporting references**

1. Collins JT, Dunnick WA. Germline transcripts of the murine immunoglobulin gamma 2a gene: structure and induction by IFN-gamma. Int Immunol. 1993 Aug;5(8):885-91. PubMed PMID: 8398983.

2. Delphin S, Stavnezer J. Characterization of an interleukin 4 (IL-4) responsive region in the immunoglobulin heavy chain germline epsilon promoter: regulation by NF-IL-4, a C/EBP family member and NF-kappa B/p50. J Exp Med. 1995 Jan 1;181(1):181-92. PubMed PMID: 7807002; PubMed Central PMCID: PMC2191820.

3. Gerondakis S, Gaff C, Goodman DJ, Grumont RJ. Structure and expression of mouse germline immunoglobulin gamma 3 heavy chain transcripts induced by the mitogen lipopolysaccharide. Immunogenetics. 1991;34(6):392-400. PubMed PMID: 1748487.

4. Gerondakis S. Structure and expression of murine germ-line immunoglobulin epsilon heavy chain transcripts induced by interleukin 4. Proc Natl Acad Sci U S A. 1990 Feb;87(4):1581-5. PubMed PMID: 2106137; PubMed Central PMCID: PMC53519.

5. Liao F, Birshtein BK, Busslinger M, Rothman P. The transcription factor BSAP (NF-HB) is essential for immunoglobulin germ-line epsilon transcription. J Immunol. 1994 Mar 15;152(6):2904-11. PubMed PMID: 8144891.

6. Lin YC, Stavnezer J. Regulation of transcription of the germ-line Ig alpha constant region gene by an ATF element and by novel transforming growth factor-beta 1-responsive elements. J Immunol. 1992 Nov 1;149(9):2914-25. PubMed PMID: 1401921.

7. Lundgren M, Larsson C, Femino A, Xu M, Stavnezer J, Severinson E. Activation of the Ig germ-line gamma 1 promoter. Involvement of C/enhancer-binding protein transcription factors and their possible interaction with an NF-IL-4 site. J Immunol. 1994 Oct 1;153(7):2983-95. PubMed PMID: 8089482.

8. Lutzker S, Alt FW. Structure and expression of germ line immunoglobulin gamma2b transcripts. Mol Cell Biol. 1988 Apr;8(4):1849-52. Erratum in: Mol Cell Biol 1988 Oct;8(10):4585. PubMed PMID: 3132612; PubMed Central PMCID: PMC363353.

9. Mao CS, Stavnezer J. Differential regulation of mouse germline Ig gamma 1 and epsilon promoters by IL-4 and CD40. J Immunol. 2001 Aug 1;167(3):1522-34. PubMed PMID: 11466373.

10. Park SR, Seo GY, Choi AJ, Stavnezer J, Kim PH. Analysis of transforming growth factor-beta1-induced Ig germ-line gamma2b transcription and its implication for IgA isotype switching. Eur J Immunol. 2005 Mar;35(3):946-56. PubMed PMID: 15688346.

11. Qiu G, Stavnezer J. Overexpression of BSAP/Pax-5 inhibits switching to IgA and enhances switching to IgE in the I.29 mu B cell line. J Immunol. 1998 Sep 15;161(6):2906-18. PubMed PMID: 9743352.

12. Radcliffe G, Lin YC, Julius M, Marcu KB, Stavnezer J. Structure of germ line immunoglobulin alpha heavy-chain RNA and its location on polysomes. Mol Cell Biol. 1990 Jan;10(1):382-6. PubMed PMID: 2152964; PubMed Central PMCID: PMC360760.

13. Rothman P, Chen YY, Lutzker S, Li SC, Stewart V, Coffman R, Alt FW. Structure and expression of germ line immunoglobulin heavy-chain epsilon transcripts: interleukin-4 plus lipopolysaccharide-directed switching to C epsilon. Mol Cell Biol. 1990 Apr;10(4):1672-9. PubMed PMID: 2157139; PubMed Central PMCID: PMC362272.

14. Rothman P, Lutzker S, Gorham B, Stewart V, Coffman R, Alt FW. Structure and expression of germline immunoglobulin gamma 3 heavy chain gene transcripts: implications for mitogen and lymphokine directed class-switching. Int Immunol. 1990;2(7):621-7. PubMed PMID: 2126196.

15. Seo GY, Park SR, Kim PH. Analyses of TGF-beta1-inducible Ig germ-line gamma2b promoter activity: involvement of Smads and NF-kappaB. Eur J Immunol. 2009 Apr;39(4):1157-66. doi: 10.1002/eji.200838732. PubMed PMID: 19266485.

16. Shen CH, Stavnezer J. Activation of the mouse Ig germline epsilon promoter by IL-4 is dependent on AP-1 transcription factors. J Immunol. 2001 Jan 1;166(1):411-23. PubMed PMID: 11123319.

17. Shi MJ, Park SR, Kim PH, Stavnezer J. Roles of Ets proteins, NF-kappa B and nocodazole in regulating induction of transcription of mouse germline Ig alpha RNA by transforming growth factor-beta 1. Int Immunol. 2001 Jun;13(6):733-46. PubMed PMID: 11369700.

18. Shi MJ, Stavnezer J. CBF alpha3 (AML2) is induced by TGF-beta1 to bind and activate the mouse germline Ig alpha promoter. J Immunol. 1998 Dec 15;161(12):6751-60. PubMed PMID: 9862705.

19. Ström L, Lundgren M, Severinson E. Binding of Ikaros to germline Ig heavy chain gamma1 and epsilon promoters. Mol Immunol. 2003 Mar;39(13):771-82. PubMed PMID: 12617992.

20. Xu M, Stavnezer J. Structure of germline immunoglobulin heavy-chain gamma 1 transcripts in interleukin 4 treated mouse spleen cells. Dev Immunol. 1990;1(1):11-7. PubMed PMID: 1726553; PubMed Central PMCID: PMC2275814.
