# Supplementary material for: Developmental regulation of DNA cytosine methylation at the immunoglobulin heavy chain constant locus
Source: PLoS Genet. 2019 Feb 19;15(2):e1007930. doi: 10.1371/journal.pgen.1007930 (PMC6380546; doi:10.1371/journal.pgen.1007930)
Supplement: S1 Table — (PDF) [file pgen.1007930.s007.pdf]

S1 Table. Primers used in this study

Region	Primer	Converted primer	Amplicon	Tm	Native sequence
• ***Promoter regions***					
Iγ3 Promoter	**Me1-Ig3F**	TTATTAAAGTTGGAAAGTTGGAAGT	221bp	60°C	TCATCAAAGCTGGAAAGTTGGAAGC
	**Me1-Ig3R**	CTCAAAAAAATCTATTTATTATAACCCC			GGGGGTTACAATAAATAGATTCCCTTGAGTCTC
	**Me2-Ig3F**	TGGGTTGTAATTTTTAAGAGGAAAG	242bp	62°C	TGGGTTGCAACTCCTAAGAGGAAAG
	**Me2-Ig3R**	AAACCCCCTCCACAATCTAATAT			ATACCAGACTGTGGAGGGGGTCT
	**Me4-Ig3F**	AGATTTTTTGAGTTTTAGAAGAGATTTTA	140bp	60°C	AGATTCCCTTGAGTCTCAGAAGAGACTCCA
	**Me4-Ig3R**	TCAAATCACCAAATAAATTCCTAAC			GCCAGGAACCTATCTGGTGATTTGA
	**Me5-Ig3F**	TTTTTTTTGGGTTTTAAGGGTATTATT	172bp	58°C	TCTCCCCTGGGTCTCAAGGGTACTATC
	**Me5-Ig3R**	CACACCCATCTCTCTACACAAACTA			TAGCCTGTGCAGAGAGATGGGTGTG
Iγ1 Promoter	**Me1-Ig1F**	GTTTTTGGATTAGTTGTTTAGGGG	265bp	56°C	GCTTCTGGACCAGCTGTCCAGGGG
	**Me1-Ig1R**	ATAACTATTAAAAACTCAAAAAACCTAAAA			TCTCAGGTCCTCTGAGCTCTCAACAGTTAC
	**Me2-Ig1F**	TTTAATTTTGTGTTTTGGGTTTTAGTT	207bp	56°C	CCTAATTCTGTGTTCTGGGTCTCAGCC
	**Me2-Ig1R**	TAAAAATCCAAAAAAATATAAAAACCT			AGGCCCTCATATCCTCCTGGATCCCCA
	**Me3-Ig1F**	TTTTTGTTTGGAAATTTAGGTAGAG	120bp	56°C	CCTCTGCTTGGAAACTCAGGCAGAG
	**Me3-Ig1R**	TCCTTATTAATACTATCTCAAAAAAACC			GGTTTTCTTGAGATAGCACCAACAAGAT
	**Me4-Ig1F**	GGGTAGGATTAAAATAGGAATAGAGA	205bp	60°C	GGGCAGGACCAAAACAGGAACAGAGA
	**Me4-Ig1R**	ACTCTTTAACACAAAAACTTCCACTAC			GCAGTGGAAGCCTCTGTGTTAAAGAGT
	**Me5-Ig1F**	AAAAATACTCTTAAATATCTCCTCCC	236bp	56°C	CCTGGTGTCAACTAGGCAGGCCCTG
	**Me5-Ig1R**	TTTGGTGTTAATTAGGTAGGTTTTG			GGGAGGAGATATCCAAGAGCATTCTT
	**Me6-Ig1F**	AGGGATTTTTAAAGGTTTAGGTGTATAG	137bp	60°C	AGGGACCCCCAAAGGCCCAGGTGCACAG
	**Me6-Ig1R**	AAATCCAAACCTAACTTAAATTACTTCATA			CATGAAGTAATCTAAGTCAGGTTTGGACTC

Iγ2b Promoter	**Me1-Ig2bF**	GAAAGAGGAATTTTAGGAAGTGTTT	233bp	60°C	GAAAGAGGAACTTCAGGAAGTGTCC
	**Me1-Ig2bR**	ACTCTCTCCAATCTCTCAACTAACC			GGTCAGCTGAGAGACTGGAGAGAGC
	**Me2-Ig2bF**	TTAGGAATATTGGGTTGAGATAATTAG	122bp	56°C	CCAGGAACACTGGGTTGAGACAACTAG
	**Me2-Ig2bR**	AAAAAAAACAAAAAAAATCCAAA			TCTGGACTCCCCCTGCTCTCCCC
	**Me5-g1g2bF**	ATGGGGGTGTAATAAGGGAGTAT	225bp	62°C	ATGGGGGTGTAATAAGGGAGTAT
	**Me5-g1g2bR**	CCTACTAAACTATTTATATCCCACAACTTC			CCTACTAAACTATTTATATCCCACAACTTC
	**Me6-g1g2bF**	TTAATTTTGTAGAGTGTGATAGAGG	123bp	58°C	TTAATTTTGTAGAGTGTGATAGAGG
	**Me6-g1g2bR**	CAAAATCTCTACTAAAACCCAAAA			CAAAATCTCTACTAAAACCCAAAA
Iγ2a Promoter	**Me1-Ig2aF**	TAGTGATGGGTATATAAGAGAGGGAGTA	133bp	62°C	CAGTGATGGGCACATAAGAGAGGGAGCA
	**Me1-Ig2aR**	CCAAAAACCTTACCTAATCCTCTAAA			CTTAGAGGACCAGGCAAGGCCTCTGG
	**Me2-Ig2aF**	TTTGTAAGAATTAGGGATTAGAATTGTG	230bp	53°C	CTTGTAAGAACCAGGGATCAGAATTGTG
	**Me2-Ig2aR**	ACAACAAATCAAATATTAAAAAAAA			TTCCCCTCAACACCTGACTTGCTGC
	**Me3-Ig2aF**	AAGTAAAAAGAGTAGTAGTTTTTTTTA	284bp	58°C	AAGCAAAAAGAGCAGCAGTCTTCTTCA
	**Me3-Ig2aR**	CTAATTAATCTATATATATCAACAACTTTAA			CTAAAGCTGCTGACACATACAGACAACCAG
	**Me4-Ig2aF**	TTTTTTTAATATTTGATTTGTTGTATTATG	140bp	56°C	TCCCCTCAACACCTGACTTGCTGCACTATG
	**Me4-Ig2aR**	ACCCAACTTAAATACCAACCTCTAC			GCAGAGGTTGGTACCTAAGCTGGGT
Iε Promoter	**Me1-IεF**	TTGGGGTAGTAGTTAGATTTTTTTT	164bp	56°C	TTGGGGTAGCAGTCAGACCTTTCCC
	**Me1-IεR**	TTCCCTTAAATAACTCCTTCACTCA			TGAGTGAAGGAGCCACCCAAGGGAA
	**Me2-IεF**	TTGTTTGGTTTTGTTATTATTAGGAAG	152bp	62°C	CCTGCCTGGCTCTGCCACTATCAGGAAG
	**Me2-IεR**	CATCCCTTAAACCCTAACTACTCAA			CTGAGCAGCCAGGGTCCAAGGGATG
	**Me3-IεF**	TGAAGGGTTGTTAAAGGAATAGAAA	191bp	62°C	TGAAGGGCTGCCAAAGGAACAGAAA
	**Me3-IεR**	CACACCTACACACTCTAATCAAAACTC			GAGCCTTGACCAGAGTGTGCAGGTGTG
	**Me4-IεF**	AAAGGGAATTTTTAAGGTTGTTAAG	205bp	56°C	AAAGGGAACTTCCAAGGCTGCTAAG
	**Me4-IεR**	AATCCTCTAATAAATAAATAACTAACCAAA			TCTGGCCAGCCACTCACTTATCAGAGGACC

	**Me1-IaF**	TTTAGTTTTGAATTTTTATTGATTAAGTTA	148bp	60°C	TTCAGCCTTGAACCTCTACTGACCAAGCCA
	**Me1-IaR**	AAAATTCTATCATCAAACCAACTA			TAGTTGGCCTGATGACAGAACTTC
	**Me2-IaF**	ATGGTTTATGTGGTTAGATATATTTGTTTT	292bp	62°C	ATGGCCCATGTGGTCAGACACACCTGTCTC
	**Me2-IaR**	TAATCCCAACTAAAAATCCATTCTC			GAGAATGGATCTTCAGTTGGGACCA
**Iα Promoter**	**Me3-IaF**	TTAATAGTATTGAGTAGGTGGGTTT	247bp	58°C	TCAACAGCATTGAGCAGGTGGGTCC
	**Me3-IaR**	AAAAAACTAAATTTCATCCATTTTT			AAAAATGGATGAAACCCAGCTTCCT
	**Me1-2IaF (ectopic)**	GAGAATTTTAGTTTTGATTTGTGTGTTT	299bp	62°C	GAGAACTCCAGCTCTGACTTGTGTGCCC
	**Me1-2IaR**	AAATCCCTCACCCACTTCCTA			CAGGAAGTGGGTGAGGGACCC
	**Me2-2IaF (endogenous)**	TTTTTTTTATTTTTAATTGTTTTTTTT	266bp	60°C	TCCCTTCTACTCTTAATTGTCCTCTCT
	**Me2-2IaR**	CTAAATAAAATCCCTCACCCACTT			AAGTGGGTGAGGGACCCCACCTAG

- ***Regulatory elements***

	**Me1deltaF**	TGATTTGGGATTGGAATAGATATTT	247bp	61°C	TGATCTGGGACTGGAACAGATACTC
	**Me1deltaR**	AACACTTCTCATACAAAAATAAAACTACC			GGCAGCCCCATCCCTGTATGAGAAGTGCC
	**Me2deltaF**	TTTATTTTTGTATGAGAAGTGTTAGTTGTT	290bp	58°C	CCCATCCCTGTATGAGAAGTGCCAGCTGCC
	**Me2deltaR**	CAATTTCTAAAAAAATCCAACCTACA			TGCAGGCTGGACCTCCCCAGAAACTG
**δ-γ3 region**	**Me3deltaF**	TAGGGGAATGTTAGGGTTAAGAAGT	223bp	61°C	CAGGGGAATGCCAGGGCTAAGAAGT
	**Me3deltaR**	TTCCACTCACCAAAAATCCTATAAT			ATCATAGGACCTCTGGTGAGTGGAA
	**Me5deltaF**	TAGGTTGTTTTTGGGTTTGTG	216bp	61°C	CAGGCTGCCTCTGGGTCTGTG
	**Me5deltaR**	TAAACCATAAAAACCAAATTCCC			GGGAATTTGGTCCTTATGGTCCA
	**Me7deltaF**	AGGGAGGAATGTATATAGTTTTTTA	208bp	56°C	AGGGAGGAATGTATACAGCTCTTCA
	**Me7deltaR**	CCAACTACAATTTAATCTATCATTAT			ACAATGATAGACCAAACTGCAGCTGG
	**Me4g1g2bF**	TAGAGTAGGTTTGAGTTTTTGTTTT	160bp	54°C	CAGAGCAGGCCTGAGCCCTT
	**Me4g1g2bR**	AAATCATCCTTATCATATAAATTAC			GCAACCCACATGACAAGGATGACT
**γ1-γ2b region**	**Me5-g1g2bF**	ATGGGGGTGTAATAAGGGAGTAT	225bp	62°C	ATGGGGGTGTAATAAGGGAGTAT
	**Me5-g1g2bR**	CCTACTAAACTATTTATATCCCACAACTTC			CCTACTAAACTATTTATATCCCACAACTTC
	**Me6-g1g2bF**	TTAATTTTGTAGAGTGTGATAGAGG	123bp	58°C	TTAATTTTGTAGAGTGTGATAGAGG
	**Me6-g1g2bR**	CAAAATCTCTACTAAAACCCAAAA			CAAAATCTCTACTAAAACCCAAAA

5'hs1RI region	**Me1NLR-F**	TTTTTAGTAGTATTTTGTTTTTTATATATG	251bp	54°C	CCCTCAGCAGCACCCTGCTCTTCACACATG
	**Me1NLR-R**	CACCTAAAATAACCTATACCCTTATCC			GGACAAGGGCATAGGCCACTCCAGGTG
	**Me2NLR-F**	ATTTTTATAGGGAGAGGGGGTAGA	240bp	62°C	ATCCCCACAGGGAGAGGGGGCAGA
	**Me2NLR-R**	AAACCAACCCTACTATCCTCAAATT			AACCTGAGGACAGCAGGGTTGGCTC
core Eμ	**EμBC-F2**	AAATGTGAGAGGGTTTTTAAGTATTT	665bp	60°C	AAATGTGAGAGGGTTTTCAAGTACTC
	**EμBC-R2**	CCAATAATCAAAAAAAAAAAATAATAAAA			CTCTATTATTCTTCCCTCTGATTATTGG
	**129EμBC-FN2**	AGGTTTTGTTTGTGTAGAATTGATATTAT	520bp	60°C	AGGTCTTGTTTGTGTAGAACTGACATTAC
	**EμBC-RN2**	CAATTTCTTACATAACCTAATTTTAAAAATA			ATTTTCAAAATTAGGTTATGTAAGAAATTG

- ***Constant exons***

Cγ3 exons	**Me1Cg3-Fw**	GGTTTTTGGTTGTAGTGATATATTTG	178bp	60°C	GGTCCCTGGCTGCAGTGACACATCTG
	**Me1Cg3-R**	TACAATCACCAAACTACTAAAAAAATAAAA			TTCTATTCCCTCAGCAGCTTGGTGACTGTA
	**Me2Cg3-Fw**	TTGGTAATATTTTGGGTGGATTATT	259bp	61°C	CTGGTAACATCTTGGGTGGACCATC
	**Me2Cg3-R**	TTACCCCTCATCCAATCCTAATAC			GCACCAGGACTGGATGAGGGGCAA
	**Me3Cg3-Fw**	GAATTGAATGAGATTTGTGTTGAG	130bp	55°C	GAACTGAATGAGACCTGTGCTGAG
	**Me3Cg3-R**	TAACCTTAAAAAAAATAACAAAAAC			CCTCTGTCACCCTCTTCAAGGTCA
Cγ1 exons	**Me1Cg1-Fw**	ATTTTGAGTAGTTTAGTGATTGTTTTTTTT	109bp	57°C	ACTCTGAGCAGCTCAGTGACTGTCCCCTCC
	**Me1Cg1-R**	CACCAATTTTCTTATCCACCTTAATAC			GCACCAAGGTGGACAAGAAAATTGGTG
	**Me2Cg1-Fw**	ATTATTATTTTGATTTTTAAGGTTA	146bp	55°C	ACCATTACTCTGACTCCTAAGGTCA
	**Me2Cg1-R**	AAAATACTATTAAACTACTCCTCC			GGAGGAGCAGTTCAACAGCACTTT
Cγ2b exons	**Me1Cg2b-F**	GAGGATAAGTTATGTATAAATTTATTTTTA	194bp	52°C	GAGGATAAGCCATGTACAAATCCATTTCCA
	**Me1Cg2b-R**	TATTCACAAACCAACTAATCTAAA			TCCAGATCAGCTGGTTTGTGAACA
Cα exons	**Me1Ca-F**	TTTTGTTAAGTGATTTAGTGATAAT	129bp	52°C	CTCTGTCAAGTGACCCAGTGATAAT
	**Me1Ca-R**	AAACCAAAACAAATAAAAAATTTA			TAAACTTCCCACCTGCCCTGGCCT
	**Me2Ca-F**	ATTTTTTATTTGTTTTGGTTTTTGG	192bp	57°C	ACTTCCCACCTGCCCTGGCCTCTGG
	**Me2Ca-R**	AAAACTTATCCCACCCCTAACTAAC			GTCAGCTAGGGGTGGGATAAGTCCT
