# Supplementary material for: Developmental regulation of DNA cytosine methylation at the immunoglobulin heavy chain constant locus
Source: PLoS Genet. 2019 Feb 19;15(2):e1007930. doi: 10.1371/journal.pgen.1007930 (PMC6380546; doi:10.1371/journal.pgen.1007930)
Supplement: S2 Table — Localization of the converted primers within the sequence of the mouse IgH locus (129S1 strain. GenBank accession number: AJ851868.3). (PDF) [file pgen.1007930.s008.pdf]

S2 Table. Localization of the converted primers within the sequence of the mouse *IgH* locus (129S1 strain. GenBank accession number: AJ851868.3)

- **δ - γ3 intergenic region:**

Me1delta (3’δ1): 1.449.014 – 1.449.260
Me2delta (3’δ1): 1.449.238 – 1.449.527
Me3delta (3’δ2): 1.457.768 – 1.457.990
Me5delta (3’δ3): 1.464.571 – 1.464.786
Me7delta (3’δ3): 1.464.983 – 1.465.190

- **Iγ3 promoter region:**

Me1Ig3: 1.478.045 – 1.478.266
Me4Ig3: 1.478.250 – 1.478.389
Me5Ig3: 1.479.709 – 1.479.880
Me2Ig3: 1.479.908 – 1.480.149

- **Cγ3 constant gene:**

Me1Cg3: 1.485.393 – 1.485.570
Me2Cg3: 1.486.165 – 1.486.423
Me3Cg3: 1.488.379 – 1.488.508

- **Iγ1 promoter region:**

Me1Ig1: 1.505.944 – 1.506.208
Me2Ig1: 1.506.357 – 1.506.563
Me3Ig1: 1.507.034 – 1.507.153
Me6Ig1: 1.507.290 – 1.507.426
Me4Ig1: 1.507.592 – 1.507.796
Me5Ig1: 1.507.815 – 1.508.050

- **Cγ1 constant gene:**

Me1Cg1: 1.521.987 – 1.522.095
Me2Cg1: 1.522.640 – 1.522.785

- **γ1 - γ2b intergenic region:**

Me4g1g2b (3’γ1E): 1.530.891 – 1.531.050
Me7g1g2b (5’γ2bE): 1.533.791 – 1.533.999
Me8g1g2b (5’γ2bE): 1.534.131 – 1.534.240
Me5g1g2b: 1.534.575 – 1.534.799
Me6g1g2b: 1.535.203 – 1.535.325

- **Iγ2b promoter region:**

Me2Ig2b: 1.537.253 – 1.537.374
Me1Ig2b: 1.537.401 – 1.537.633

- **Cγ2b constant gene:**

Me1Cg2b: 1.543.768 – 1.543.961

- **Iγ2a promoter region:**

Me1Ig2a: 1.551.159 – 1.551.291
Me2Ig2a: 1.552.149 – 1.552.372
Me4Ig2a: 1.552.349 – 1.552.488
Me3Ig2a: 1.552.882 – 1.553.165

- **Iε promoter region:**

Me1Iε: 1.568.967 – 1.569.130
Me2Iε: 1.569.810 – 1.569.961
Me3Iε: 1.570.551 – 1.570.741
Me4Iε: 1.570.808 – 1.571.012

- **Iα promoter region:**

Me1Iα: 1.581.942 – 1.582.089
Me2-2Iα: 1.582.891 – 1.583.156
Me2Iα: 1.583.035 – 1.583.326
Me3Iα: 1.583.368 – 1.583.614

- **Cα constant gene:**

Me1Cα: 1.588.740 – 1.588.868
Me2Cα: 1.588.848 – 1.589.039

- **5’hs1RI region:**

Me1NLR: 1.591.010 – 1.591.260
Me2NLR: 1.591.747 – 1.591.986
